# Cognitive abilities affect decision errors but not risk preferences: A meta-analysis

**DOI:** 10.3758/s13423-021-02053-1

**Published:** 2022-03-30

**Authors:** Tehilla Mechera-Ostrovsky, Steven Heinke, Sandra Andraszewicz, Jörg Rieskamp

**Affiliations:** 1 School of Psychology, University of New South Wales Sydney, Sydney, 2052 Australia; 2 Department of Psychology, Center for Economic Psychology, University of Basel, Basel, Switzerland; 3 Chair of Cognitive Science, ETH Zurich, Zürich, Switzerland; 4 Singapore-ETH Centre, Future Resilient Systems, CREATE campus, 1 CREATE Way, #06-01 CREATE Tower Singapore, 138602 Singapore

**Keywords:** Value-based decisions, Risk preferences, Cognitive ability, Raven’s matrices, Cognitive Reflection Test, Multiple price list, Meta-analysis

## Abstract

When making risky decisions, people should evaluate the consequences and the chances of the outcome occurring. We examine the *risk-preference hypothesis*, which states that people’s cognitive abilities affect their evaluation of choice options and consequently their risk-taking behavior. We compared the risk-preference hypothesis against a parsimonious *error hypothesis*, which states that lower cognitive abilities increase decision errors. Increased decision errors can be misinterpreted as more risk-seeking behavior because in most risk-taking tasks, random choice behavior is often misclassified as risk-seeking behavior. We tested these two competing hypotheses against each other with a systematic literature review and a Bayesian meta-analysis summarizing the empirical correlations. Results based on 30 studies and 62 effect sizes revealed no credible association between cognitive abilities and risk aversion. Apparent correlations between cognitive abilities and risk aversion can be explained by biased risk-preference-elicitation tasks, where more errors are misinterpreted as specific risk preferences. In sum, the reported associations between cognitive abilities and risk preferences are spurious and mediated by a misinterpretation of erroneous choice behavior. This result also has general implications for any research area in which treatment effects, such as decreased cognitive attention or motivation, could increase decision errors and be misinterpreted as specific preference changes.

When facing risky decisions, such as selecting one of several treatments for a severe disease or choosing stock investments, people evaluate the potential consequences of their decisions. For example, a physician needs to consider possible treatment outcomes, such as successful recovery from a disease, side effects, or fatality. An investor analyses potential profits and losses from potential stock investments. These decisions rely on cognitive processes such as option valuation, action selection, outcome valuation, and potentially learning about the outcomes (Rangel et al., 2008). The cognitive processes strongly rely on cognitive abilities and individual preferences. A plethora of research has investigated the effect of cognitive abilities on decision making across various domains (for a review, see Dohmen et al., 2018). However, previous literature provides inconsistent evidence on whether cognitive abilities affect people’s risk preferences. Some studies have shown a negative correlation between cognitive abilities and risk-averse preferences, while others have indicated a positive correlation. Thus, the mechanisms underlying the relation between one’s cognitive abilities, individual risk preferences,^1^ and risk taking remain unclear.

The *risk-preference hypothesis* states that there is a systematic link between people’s cognitive abilities and their risk preferences, because cognitive abilities affect the evaluation of risky options and, consequently risk-taking behavior (Frederick, 2005). Different rationales underlie this hypothesis. First, it could be that people with higher cognitive abilities are confident that they can evaluate the costs and benefits of risky decisions accurately and are therefore more willing to take risk and are more risk seeking. In contrast, people with lower cognitive abilities are less confident that they evaluate the cost and benefits of risky decisions accurately and fear that they make unreasonable choices, so that they are in general more risk averse. This leads to a general negative correlation between cognitive abilities and risk averse preferences. Second, it could be that people with higher cognitive abilities can evaluate choice options accurately according to their risk and rewards (i.e., variance and expected value), and decide consistently with their latent risk preferences. In contrast, people with lower cognitive abilities rely on less demanding heuristics, so that their risk-taking behavior might not correspond with their latent risk preference but could be more risk averse or more risk loving depending on the used heuristic and the task characteristics. If their behavior is biased toward risk aversion, a negative correlation between cognitive abilities and the observed risk-averse behavior results. In contrast, if it is biased toward risk seekingness, a positive correlation result. In other words, depending on the heuristics used by people with lower cognitive abilities, one can expect positive or negative correlations between people’s cognitive abilities and the observed risk-taking behavior. However, the variability in correlations due to the use of different heuristics in tasks with identical characteristics should not correlate with the effect of these characteristics on random choice behavior, an alternative explanation to the possible variations in correlations, which we describe below. In other words, whether a task is biased toward risk-averse behavior so that random choice behavior leads to a risk-averse classification should not affect the correlation due to the use of different heuristics*.*

The alternative *error hypothesis* rejects the notion of a systematic link between people’s cognitive abilities and their cognitive latent risk preferences. Instead, this hypothesis states that a negative correlation between people’s cognitive abilities and the number of decision errors exists (Andersson et al., 2016; Olschewski et al., 2018). Naturally, the predicted errors depend on the specific error theory assumed. For instance, sequential sampling models assume that when people accumulate evidence supporting one choice option or another, the evidence entails some noise (Rieskamp, 2008). Following the error hypothesis, this noise level should be higher for people with lower as compared with higher cognitive abilities. However, for the sake of simplicity, in the present work we assume a “constant” or “trembling hand” error theory (Loomes et al., 2002): Accordingly, people follow a specific decision strategy, but with a constant probability they make errors so that one of the available choice options is randomly chosen with equal probability. According to the error hypothesis, people with lower compared with higher cognitive abilities should have a higher probability of a trembling hand error. Therefore, the trembling hand error implies more unsystematic choices for people with lower cognitive abilities. In the extreme case, people with a maximum probability of a trembling hand error would always choose each of the available choice options with equal probability, which we call *random choice behavior*.

A link between cognitive abilities and decision errors can also lead to an empirical correlation between cognitive abilities and people’s elicited risk preferences. The rationale here is that most risk-preference-elicitation tasks posit that people are in general risk averse, resulting in most tasks having a higher resolution for measuring risk aversion than risk seekingness. Accordingly, in most tasks, the riskier option will predominantly offer a higher EV than the safer option. Although a higher resolution for measuring risk aversion appears sensible, it has the disadvantage that someone with a maximum trembling hand error (i.e., showing random choice behavior) will necessary be classified as being risk averse. In general, the higher the trembling hand error probability, the more likely it is that this person will be assessed as risk averse and consequently, people with lower cognitive abilities, potentially having a higher trembling hand error, will appear as being more risk averse. For example, consider a risk-preference-elicitation task with 10 two-alternative forced choices where for seven choices the riskier option has a higher EV. A risk-averse decision maker now has seven decisions in which to express their degree of risk aversion, while a risk-seeking decision maker can express their degree of risk seekingness only in the remaining three choices. According to the error hypothesis, a person with low cognitive abilities makes more trembling hand errors. In the most extreme case, namely, with the highest trembling hand error, the person will choose the less risky choice option on average five times and will be incorrectly classified as being risk averse.

We explored the relationship between cognitive abilities and risk preferences by conducting a systematic meta-analysis. The results of the meta-analysis support the error hypothesis as an explanation for the inconsistencies found in the previous literature. These findings shed light on how risk-preference-elicitation task architectures can lead to a systematic bias in the measurement of people’s risk preferences when not controlling for people’s decision errors.

## The puzzle of cognitive abilities and risk taking

While some studies have found a positive correlation between cognitive abilities and risk aversion (i.e., Agarwal & Mazumder, 2012; Burks et al., 2009; Dohmen et al., 2007), others have reported a negative one (i.e., Li et al., 2015; Rustichini, 2015). A recent meta-analysis by Lilleholt (2019) investigated the puzzle of the link between cognitive abilities and risk taking and concluded that there is only a small negative correlation between cognitive abilities and risk taking in the gain domain and a null effect in the loss or mixed domain. Numerous researchers have challenged the existence of a causal link between cognitive abilities and risk aversion (e.g., Andersson et al., 2016). Arguing in line with the error hypothesis, they have claimed that the variability in previous findings is simply driven by errors associated with the decision process (e.g., lack of attention) and further, that task architecture could lead to a biased interpretation of the observed risk-taking behavior. In sum, if the error hypothesis is the driver behind the mixed results, a more general conclusion can be drawn: Different reported correlations involving individuals’ preferences could be better explained by an increase in decision errors. Given the mixed results and interpretations in the realm of cognitive abilities and risk taking, it appears necessary and appropriate to provide a detailed look into the existing literature and to address the question of what might cause the potential variability in the results.

Eliciting and interpreting risk preferences accurately is crucial for both practitioners and researchers in economics and psychology. For instance, financial institutions are required to adapt their investment options to the risk preferences of their clients.^2^ Researchers rely on estimates of individual risk preferences when taking these preferences into account as control variables in their data analysis (e.g., Charness et al., 2013). Consequently, researchers have put great effort into establishing reliable methods to elicit risk preferences (for a review, see Charness et al., 2013; Crosetto & Filippin, 2016; Dave et al., 2010). One group of these methods employs various behavioral tasks, all measuring individual willingness to take risks. The tasks differ in their architecture: the number of choices, the (graphical) presentation of options, the domain of the possible outcomes (e.g., gain, loss, or mixed outcome), and the framing of the decisions. Although, in principle, these architectural differences should not affect the measured preferences, it has been shown that different elicitation methods lead to different measurement results (Frey et al., 2017)^3^.

Many behavioral tasks designed to elicit risk preferences consist of a set of two-alternative forced choices between gambles, where one choice option is riskier than the other. A widely used format among such tasks is the so-called multiple price list (MPL) method. Table 1 illustrates an example used by Dohmen et al. (2011). This MPL consists of 20 decisions between a sure-payment option and a gamble. While the gamble remains the same for all decisions, the payment of the sure option increases. Therefore, the sure option becomes more attractive, relative to the gamble, as the decision maker moves down the list. Compared with other risk-preference-elicitation methods, this exemplary MPL architecture is considered relatively simple to understand and fast to administer (Chapman et al., 2018). Nevertheless, such risk-preference-elicitation tasks do require some degree of cognitive abilities to complete (Charness et al., 2017; Olschewski et al., 2018). For instance, in the example in Table 1, at Row 16 the EV of the gamble equals the (expected) value of the sure option. Consequently, as the EV of the gamble is higher than the sure option for Rows 1 to 15, a risk-neutral decision maker should choose the gamble in these rows and switch to the sure option in Rows 17 to 20. Note that on Row 16, a risk-neutral decision maker should be indifferent about the options. Accordingly, a risk-averse decision maker is expected to switch to the sure option earlier than a risk-neutral decision maker, whereas a risk-seeking decision maker is expected to switch later. It requires some cognitive effort to follow this rationale.

**Table 1:** An illustrative example of a multiple price list

Decision no.	Sure payment	Gamble
1	$0 for sure	50%, $300 and 50%, $0
2	$10 for sure	50%, $300 and 50%, $0
3	$20 for sure	50%, $300 and 50%, $0
4	$30 for sure	50%, $300 and 50%, $0
5	$40 for sure	50%, $300 and 50%, $0
6	$50 for sure	50%, $300 and 50%, $0
7	$60 for sure	50%, $300 and 50%, $0
8	$70 for sure	50%, $300 and 50%, $0
9	$80 for sure	50%, $300 and 50%, $0
10	$90 for sure	50%, $300 and 50%, $0
11	$100 for sure	50%, $300 and 50%, $0
12	$110 for sure	50%, $300 and 50%, $0
13	$120 for sure	50%, $300 and 50%, $0
14	$130 for sure	50%, $300 and 50%, $0
15	$140 for sure	50%, $300 and 50%, $0
16	$150 for sure	50%, $300 and 50%, $0
17	$160 for sure	50%, $300 and 50%, $0
18	$170 for sure	50%, $300 and 50%, $0
19	$180 for sure	50%, $300 and 50%, $0
20	$190 for sure	50%, $300 and 50%, $0

Adapted from Dohmen et al. (2011)

### The risk-preference hypothesis

According to the risk-preference hypothesis, a correlation between cognitive abilities and risk preferences exists. The risk preference hypothesis can be motivated by different theoretical arguments why a correlation between people’s cognitive abilities and their observed risk-taking behavior exists. First, it could be that there is a correlation between people’s cognitive abilities and their latent cognitive risk preferences. People with higher cognitive abilities could be more confident in evaluating the costs and benefits of risky decisions more accurately and are therefore in general more risk-seeking than people with low cognitive abilities. In contrast, people with lower cognitive abilities are less confident that they are able to evaluate the costs and benefits of risky options accurately and fear to make unreasonable risky decisions so that they are in general more risk-averse than people with higher cognitive abilities. According to this argument, a negative correlation between people’s cognitive abilities and their latent cognitive risk-averse preferences should exist, which would also be reflected in the observed risk-taking behavior. Second, the risk preference hypothesis is often motivated by the assumption that people with high cognitive abilities apply different decision strategies for solving risky decision-making problems than people with lower cognitive abilities (Osman, 2004; Stanovich & West, 1998). Prominent theories include dual-process theories (Kahneman & Frederick, 2002; Osman, 2004; Stanovich & West, 2003) and narrow bracketing (Dohmen et al., 2011; Koch & Nafziger, 2016; Read et al., 1999). Both theories hypothesize that people with higher cognitive abilities acquire a larger amount of information and process it more analytically following normative standards, such as preferring higher EV and lower variance of possible outcomes (Cokely & Kelley, 2009; Kokis et al., 2002).

In contrast, people with lower cognitive abilities are assumed to rely on simple heuristics (Cokely & Kelley, 2009; Kokis et al., 2002). Because people with different cognitive abilities could use different decision strategies, their risk-taking behavior might also differ. For instance, the maximin and maximax rules could explain how people make decisions for the problem presented in Table 1. The maximax decision rule focuses solely on outcomes, ignoring their corresponding probabilities, so for the MPL presented in Table 1 the gamble is chosen in all instances (i.e., Rows 1–20). As a consequence, a decision maker applying this rule will be classified as very risk seeking. In contrast, a person applying the maximin strategy will choose the option with the best of the worst payoffs, which in the MPL in Table 1 means choosing the sure option in all instances. Consequently, this choice behavior will be classified as very risk averse. These examples show that depending on the applied strategy, the observed behavior will be different, affecting the measurement of people’s risk preferences. Moreover, the use of a specific risk-preference-elicitation method might trigger different strategies for people with different cognitive abilities. Consequently, the observed correlation between cognitive abilities and risk aversion can be affected in different ways.^4^ Although the correlation between people cognitive abilities and their risk-taking behavior could differ depending on the used heuristics and the characteristics of the task one would not expect that this correlation is affected by the random choice risk-taking bias described next.

### The error hypothesis

The error hypothesis states that cognitive abilities are related to decision errors. People with low cognitive abilities are more prone to errors when they process information and might make choices with less accuracy, whereas people with high cognitive abilities make more accurate decisions (e.g., Galarza & Bejarano, 2016). Similarly, when people’s cognitive abilities are challenged, for instance, by depleted cognitive resources in a dual-task manipulation, it should also lead to more decision errors (Galarza & Bejarano, 2016; Olschewski et al., 2018). Deck et al. (2021) found that cognitive load results in poorer performance on math problems and higher risk aversion (Deck et al., 2021). However, depending on the risk-preference-elicitation method, an increase in errors could be misinterpreted as high or low risk aversion, a phenomenon that depends on the specific task architecture of the elicitation method (Galarza & Bejarano, 2016; Jacobson & Petrie, 2009; Zhang et al., 2020).

To illustrate the implications of this error mechanism, suppose a decision maker chooses randomly with equal probability between two choice options in a standard risk-preference-elicitation task. This random choice behavior would, on average, lead to choosing the safe option in 50% of the cases and the risky option in the remaining 50%. This implies that, for instance, in the MPL of Dohmen et al. (2011), as presented in Table 1, random choice behavior will be classified as risk aversion, because this behavior implies choosing the risky options less frequently compared with a risk-neutral decision maker (i.e., a risk-neutral decision maker is expected to make approximately 75% risky choices). Thus, depending on the risk-preference-elicitation task and its unique architecture, different levels of risk aversion can be inferred as a result of random choice behavior. A link between cognitive abilities and the number of unsystematic errors could, therefore, be misinterpreted as a correlation between cognitive abilities and risk aversion (see also Andersson et al., 2016). This would imply that overall, the observed correlation between cognitive abilities and risk aversion is spurious.

The above-described example appears to be very realistic when considering that a common problem with the MPL is that its outcome greatly depends on the tested population. A significant fraction of respondents show multiple switching points between the two options, indicating that they do not understand the logic of the procedure (Bruner, 2017; Charness et al., 2013). In the data set of Hefti et al. (2016), around 5.7% of the 672 participants—students from the University of Zurich and the Swiss Federal Institute of Technology in Zurich—switched more than once in the MPL measuring risk preferences. Hefti et al.’s study showed a negative correlation of *r* = −.21 (*p* < .001) between participants’ cognitive abilities (measured by the number of solved Raven’s matrices) and multiple switching points (Hefti et al., 2016). That is, individuals with higher cognitive abilities were more consistent in their choices.

### The random choice risk-taking bias

If a choice set such as the exemplary MPL in Table 1 is unbalanced and biased toward more risk-averse options, decision makers with a high error probability will make many random choices and are automatically more likely to be classified as risk averse. Such a potential “bias” in the option set toward risk aversion results from the general assumption that most people are risk averse. Therefore, most risk-preference-elicitation tasks aim for higher precision in measuring risk aversion by having, predominately, choice situations in which the risky option offers a higher EV than the safe option. If we assume that most risk-preference-elicitation tasks are “biased” toward risk aversion (i.e., the task architecture is constructed such that a person behaving randomly is more likely to be classified as risk averse than risk neutral or risk seeking), then, on average, most studies will find a positive correlation between cognitive abilities and risk aversion. The direction of the bias toward a specific risk preference can be determined for any given risk-preference-elicitation method by simply inspecting the consequences of random choice behavior. We refer to this as the random choice risk-taking (RCRT) bias. To quantify the RCRT bias we determine to what extent random choice behavior (i.e., choosing with equal choice probability from all available options) leads to a specific elicited risk preference compared with a risk-neutral decision maker. The RCRT bias will affect people’s elicited risk preferences. Suppose a person has a “true” risk preference that implies a specific switching point for an MPL. This person will with some probability commit a decision error, meaning that the person will choose randomly between the two choice options due to a trembling hand error. In the case of a task with an RCRT bias towards risk aversion, this person will therefore most likely appear a bit more risk averse than the person truly is.

Following the error hypothesis, cognitive abilities affect the size of this trembling hand error; that is, for people with lower cognitive abilities, if the switching point does not coincide with the middle of the MPL, these decision makers will always have more options either above or below their true preferences, and with a considerable trembling hand error this asymmetry in the number of options leads to a biased interpretation of RCRT. As described above, we choose to measure the RCRT bias with respect to risk neutrality, as this allows for a clear reference point, seems to be the least controversial assumption, and simplifies the interpretation. A change of the reference preference to either a very risk-averse or a very risk-seeking decision maker would change the direction and thus the interpretation of the bias but would not alter the underlying problem.

Andersson et al. (2016) tested the influence of potential biases caused by an unbalanced choice set on the link between cognitive abilities and risk aversion by presenting their participants with two types of MPLs. One MPL was explicitly constructed to impose a bias towards risk seeking and the other to impose a bias towards risk aversion. The authors found that people with lower cognitive abilities showed random choice patterns more often than those with higher cognitive abilities. Consequently, participants were classified as having risk-seeking (or risk-aversion) preferences if the MPL was biased toward risk seeking (or risk aversion). The current meta-analysis goes beyond Anderson et al.’s work by examining whether the error hypothesis could help one interpret the potential link between cognitive abilities and risk preferences across a large data set of many studies.

### Additional task architecture aspects

In sum, considering the task architecture seems crucial to ensuring a flawless interpretation of people’s risk-taking behavior in a specific task. However, RCRT is only one component that defines risk-preference-elicitation task architecture. Additional characteristics of the elicitation method may guide people’s responses and should also be examined. For instance, people might perceive losses differently from gains, so the task framing may impact choice behavior (Kahneman & Tversky, 1979). Dohmen et al. (2018) suggested that the variability in previous results in the correlation between cognitive abilities and risk preferences might also be explained by whether the task environment was a loss or a gain domain. In addition, attentional processes might affect choice accuracy and thereby the observed correlation. For instance, when attention decreases with the length of a task, the task’s length should be accounted for. Furthermore, an established preference for certainty (i.e., a preference for choice options that offer sure outcomes) can lead to a shift in preferences when options with sure outcomes are available, as opposed to a presentation of two risky gambles with no sure outcomes (i.e., with outcome probabilities always <1). To investigate the impact of RCRT and other task architecture characteristics on the relation between cognitive abilities and risk aversion, we documented the relevant task characteristics and included them in our meta-analytical models. In this way, we tested which task architecture characteristics can explain the variation in previous results.

We carried out a meta-analysis of 30 studies to better understand the relationship between cognitive abilities and risk aversion. We tested two potential hypotheses—the risk-preference hypothesis, which implies that higher cognitive abilities correlate with risk taking, and the error hypothesis, which states that the observed correlations between cognitive abilities and risk taking are a consequence of random choices. As a statistical tool, the meta-analysis allowed us to quantitatively summarize existing results and weigh them with respect to their precision. Moreover, the meta-analysis allowed us to account for other important explanatory variables that can help explain the variability in previous findings.

Like our approach, a recent meta-analysis on cognitive abilities and risk aversion by Lilleholt (2019) covered a broad range of risk-preference-elicitation methods, including decisions from both experience and description. The authors reported a weak negative correlation for decisions in the gain domain and no effects in the mixed and loss domains. In light of the description–experience gap (Hertwig & Erev, 2009), these two paradigms substantially differ in the cognitive mechanisms used by decision makers and observed risk-taking results. Our meta-analysis focusses on decisions from description, which should allow for a better interpretation of the underlying cognitive mechanism explaining inconsistent results. Therefore, our meta-analysis resulted in fewer eligible studies than the study of Lilleholt (2019) but allows for an easier and direct interpretation of the results.

In the following, we first outline the methodology of our literature search and the compilation of results. We proceed to present our main findings and conclude with our interpretation of our key findings and their practical implications.

## Method

### Literature search

**Fig. 1:**
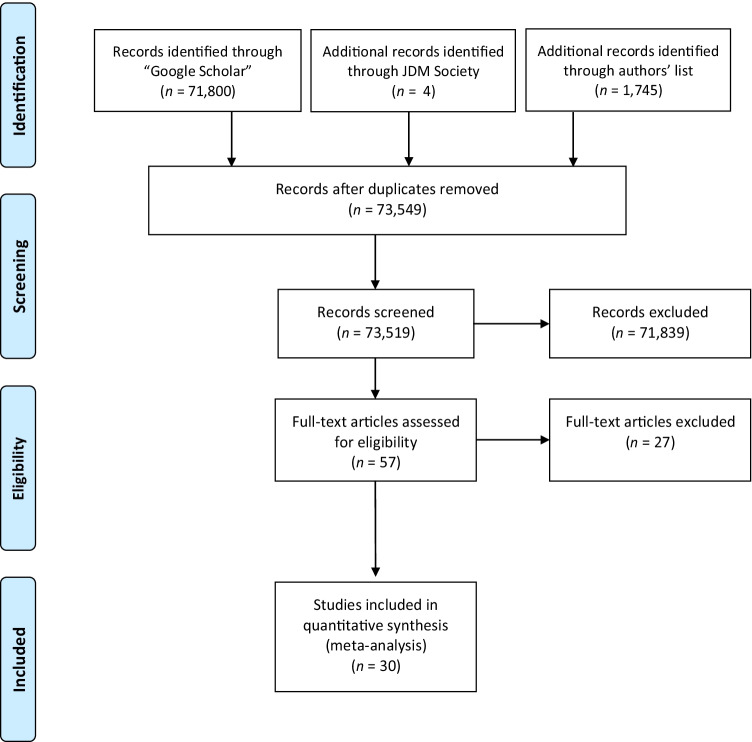
Flow chart detailing identification and screening steps and respective number of studies included in the systematic literature search and meta-analysis. *Note.* JDM Society = Society for Judgment and Decision Making

We conducted a systematic and extensive literature search using multiple methods to identify eligible research articles (see Fig. 1 for a detailed description of the process). First, we searched for literature using the search engine Google Scholar. We restricted our search to English articles published in 1990–2018 with the following keyword combinations: “IQ” AND “risk preferences”; “cognitive skills” AND “risk preferences.” Additional keyword combinations related to research fields that often include risk-preference-elicitation tasks and cognitive ability tests in their experimental routine were inspected: “aging AND risk AND cognitive ability” and “gender AND risk preferences AND cognitive ability.” Second, to include unpublished manuscripts and articles that were not easily identified with the above keywords, we posted a message requesting related published and unpublished studies to all members of an email list of the Society for Judgment and Decision Making.

We read titles and abstracts of all the obtained articles to decide if they examined a correlation between cognitive abilities and risk preferences. We next examined the eligible articles by reading the full text to ensure their relevance. Finally, for the articles that we declared eligible, we inspected their bibliography. Altogether, this process yielded 30 eligible articles (see Fig. 1). Table 2 provides the full list of all eligible studies and their corresponding characteristics.

**Table 2:** Studies included in the meta-analysis and their characteristics

Study ID	First author (year)	*N*	Elicitation method	Domain	Number of choices	Cognitive test	RCRT_bias_ (categorized)	RCRT_bias_ (continuous)
1	Frederick (2005)	3,150	MPL	Gain	8	CRT	RA	0
	Frederick (2005)	1,014	MPL	Gain	5	CRT	RL	1
	Frederick (2005)	1,366	MPL	Loss	5	CRT	RL	1
	Frederick (2005)	368	MPL	Gain	8	SAT	RA	0
	Frederick (2005)	149	MPL	Gain	5	SAT	RL	1
	Frederick (2005)	275	MPL	Loss	5	SAT	RL	1
2	Benjamin (2005)	57	MPL	Gain	5	GPA	RA	.2
	Benjamin (2005)	92	MPL	Gain	5	SAT	RA	.2
	Benjamin (2005)	92	MPL	Loss	5	SAT	RA	0
3	Oechssler et al. (2009)	563	MPL	Gain	1	CRT	RA	0
	Oechssler et al. (2009)	563	MPL	Loss	1	CRT	RL	1
4	Burks (2009)	1,000	MPL	Gain	24	IQ/Raven	RA	.17
5	Borghans et al. (2009)	327	Urn	Gain	1	CRT	RN	.5
6	Dave et al. (2010)	881	MPL_1_	Gain	6	Math/Numeracy	RA	.83
	Dave et al. (2010)	881	MPL_2_	Gain	10	Math/Numeracy	RL	.4
7	Campitelli (2010)	157	MPL	Gain	8	CRT	RA	0
	Campitelli (2010)	157	MPL	Gain	5	CRT	RL	1
	Campitelli (2010)	157	MPL	Loss	5	CRT	RL	1
8	Sousa (2010)	106	MPL	Gain	6	CRT	RA	.4
	Sousa (2010)	106	MPL	Gain	6	IQ/Raven	RA	.4
9	Dohmen et al. (2011)	452	MPL	Gain	20	IQ/Raven	RL	.75
10	Tymula (2013)	135	MPL	Gain	100	IQ/Raven	RA	.4
11	Taylor (2013)	97	HL_1_	Gain	10	CRT	RA	.4
	Taylor (2013)	97	HL_2_	Gain	10	CRT	RA	.4
12	Noussair et al. (2014)	109	MPL	Gain	5	CRT	RA	.2
13	Booth et al. (2014)	234	MPL	Gain	20	IQ/Raven	RA	.2
14	Mollerstrom (2014)	247	MPL	Gain	8	IQ/Raven	RA	.38
15	Dean (2015)	190	MPL	Gain	42	IQ/Raven	RA	0
16	Li et al. (2015)	619	MPL	Gain	16	IQ/Raven	NA_a_	NA
	Li et al. (2015)	619	MPL	Gain	16	Math/Numeracy	NA_a_	NA
17	Sinayev (2015)	1,478	HL	Gain	10	CRT	RA	.4
	Sinayev (2015)	1,478	HL	Gain	10	IQ/Raven	RA	.4
	Sinayev (2015)	1,478	HL	Gain	10	Math/Numeracy	RA	.4
18	Burks et al. (2015)	100	MPL	Gain	24	IQ/Raven	RA	.17
	Burks et al. (2015)	100	MPL	Gain	24	Math/Numeracy	RA	.17
19	Eisenberg (2018)	522	HL	Gain	10	CRT	RA	.4
	Eisenberg (2018)	522	HL	Gain	10	IQ/Raven	RA	.4
20	Noori (2016)	395	MPL	Gain	1	CRT	RA	0
	Noori (2016)	395	MPL	Loss	1	CRT	RL	1
21	Haridon (2016)	1,739	MPL	Gain	14	GPA	RN	.5
	Haridon (2016)	1,739	MPL	Loss	13	GPA	RN	.5
22	Andersson et al. (2016)	893	MPL_1_	Gain	10	CRT	RA	.2
	Andersson et al. (2016)	893	MPL_1_	Gain	10	IQ/Raven	RN	.5
	Andersson et al. (2016)	893	MPL_2_	Gain	10	CRT	RA	.2
	Andersson et al. (2016)	893	MPL_2_	Gain	10	IQ/Raven	RN	.5
23	Rustichini (2016)	130	MPL	Gain	10	IQ/Raven	RL	.66
24	Kocher et al. (2019)	80	MPL	Gain	11	CRT	RN	.45
25	Taylor (2016)	94	HL_1_	Gain	10	IQ/Raven	RA	.4
	Taylor (2016)	87	HL_2_	Gain	10	IQ/Raven	RA	.4
	Taylor (2016)	93	HL_3_	Gain	10	IQ/Raven	RA	.4
	Taylor (2016)	88	HL_4_	Gain	10	IQ/Raven	RA	.4
26	Fairley (2017)	187	MPL	Gain	10	CRT	RN	.5
	Fairley (2017)	187	MPL	Gain	10	IQ/Raven	RN	.5
27	Hefti (2016)	256	MPL	Gain	20	CRT	RA	.2
	Hefti (2016)	640	MPL	Gain	20	IQ/Raven	RA	.2
28	Corgnet (2016)	89	MPL	Gain	10	CRT	RA	.4
	Corgnet (2016)	89	MPL	Gain	10	IQ/Raven	RA	.4
29	Horn (2018)	242	Ellsberg	Gain	1	CRT	RN	0
30	Heinke (2022)	186	MPL	Gain	13	IQ/Raven	RN	.46
	Heinke (2022)	186	MPL	Gain	11	IQ/Raven	RN	.45
	Heinke (2022)	186	MPL	Gain	10	IQ/Raven	RL	.6
	Heinke (2022)	186	MPL	Gain	10	IQ/Raven	RN	.5

See Table 3 for descriptions of the risk-preference-elicitation tasks. MPL = Multiple price list. The MPL subscript numbering refers to different MPLs used in the same study. Urn = Urn Problem. HL = Holt and Laury. Ellsberg = Ellsberg Paradox Task. Gain = Elicitation method presented in the gain domain. Loss = Elicitation method presented in the loss domain. IQ/Raven = Standardized IQ tests. CRT = Cognitive Reflection Test (Frederick, 2005). Math/Numeracy = Standardized math skills or numeracy tests that are used as a proxy for intelligence tests. RA = Elicitation method has a random choice risk-taking (RCRT) bias towards risk aversion. RL = Elicitation method has an RCRT bias towards risk lovingness (i.e., risk seeking). RN = Elicitation method has no RCRT bias (i.e., bias towards risk neutrality). GPA = School mean grade point average in mathematics. SAT = a standardized test used for college admissions in the U.S. RCRT bias (continuous) column represents the proportion of options where the EV of the safe option was greater than the EV of the risky option associated with each reported effect size

^a^The authors applied a dynamic approach to the standard MPL such that the choice set was not identical for all participants

**Table 3:** Behavioral tasks included in the meta-analysis

Task	Task description
MPL (sure payment option vs. risky gamble)	A decision is made between a sure amount and a risky gamble. The list can be a series of decisions being made in the gain or loss domain.
Holt and Laury	A decision is made 10 times between two gambles with probabilities ranging from 0.1 to 0.9.
Urn Problem	A single bet is made on one of two coloured balls. Each colour is associated with a probability defining the distribution of the balls in the given urn.

MPL = Multiple Price List. Holt and Laury (Holt & Laury, 2002)

### Inclusion criteria

We included articles that complied with the following two criteria: The studies had to (a) apply a behavioral measure of risky decision-making to assess participants’ risk preferences (for a full list of the behavioral tasks included, see Table 4) and (b) report correlational results on the link between cognitive abilities and risk preferences. If any other form of association or no correlation was reported, we contacted the authors to determine the study’s inclusion eligibility (see Method, Statistical Analysis section).

**Table 4:** Moderator variables included in the meta-analysis

Moderator (Code)	Description	Examples
RCRT bias (RCRT)	The proportion of choices in which the EV of the less risky choice option is larger, smaller, or equal to the EV of the riskier one	Whenever there are fewer choices in which the EV of the less risky choice option is below the EV of the riskier choice option, a decision maker committing random error is more likely to be classified as risk averse
Cognitive ability test (CAT)	Test of cognitive ability	IQ, Raven’s matrices, CRT
Domain (Domain)	The domain in which the risk-preference-elicitation task was presented	Elicitation methods described in either the gain or the loss domain
Type of risk-preference-elicitation task (MPL)	Risk-preference-elicitation tasks consisting of a set of choices between certain and risky options vs. choices between two risky options	Multiple Price List, Urn Problem
Number of choice options (#Choices)	The number of binary choices presented to participants in the risk-preference-elicitation task	The standard HL number of choices is 10

CRT = Cognitive Reflection Test (Frederick, 2005). EV = Expected value. HL = Holt and Laury (Holt & Laury, 2002). RCRT = Random choice risk taking

### Study coding

We investigated the study characteristics documented in Table 2 to explore how they affect the variability in effect sizes among previous studies. For a list of the moderator variables and their corresponding codes, see Table 4. In this section we outline and discuss the architecture and sample-related attributes of each recorded task and provide a detailed description of the coding scheme applied. The explanatory variables we included are RCRT bias; the type of cognitive test for measuring cognitive abilities, that is, the Cognitive Reflection Test (CRT; Frederick, 2005) or a validated cognitive abilities test; the domain (i.e., tasks that include choice options with potential loss vs. tasks that incorporate a choice between two options associated with potential gains); the type of choice options (i.e., choice sets that include a sure gain vs. choice sets that include two risky options); and the number of choices in a given choice set.

#### RCRT bias (RCRT)

According to the error hypothesis, depending on the option set of the risk-preference-elicitation method, a person making random choices will be classified as risk averse, risk neutral, or risk seeking. To examine this hypothesis*,* we included a moderator variable that captures the proportion of options for which the EV of the safe option was larger than the EV of the risky option. Hence, this variable captures the degree of RCRT bias that is associated with each elicitation method. Put simply, the greater the proportion is, the greater the degree of RCRT bias. Moreover, each choice set can be categorized into one of three groups: (a) RCRT bias towards risk aversion implies more choice items in which the risky option has a higher EV than the safe option; (b) RCRT bias towards risk seeking implies more choice items in which the safe choice option has a higher EV than the risky choice option; and (c) no RCRT bias implies an equal number of choice items with a higher EV for safe and risky options. For a dummy coding of the RCRT bias we used the no-bias studies as the reference group. Because only a handful of studies applied a choice set that was bias free (i.e., Cluster no. 3), a comparison between these clusters was used to complement the analysis of the continuous variable. We therefore report the latter results in the Appendix (see Table 6).

#### Cognitive Ability Test (CAT)

The two most common ways of measuring cognitive abilities are standardized IQ tests such as the Raven’s Progressive Matrices test (Raven & Raven, 2003) and the CRT. Both measures have been particularly prevalent in psychological and economics studies investigating risk taking and decision processes. Although scores exhibited by these two cognitive ability tests correlate with each other, the tests differ in content and the individual traits and properties that they assess (Frederick, 2005; Raven & Raven, 2003; Toplak et al., 2011). While standardized cognitive ability tests are used to test fluid intelligence, the CRT is a widely used tool to measure individual differences in intuitive–analytic cognitive styles as suggested by dual-process theories (Campitelli & Gerrans, 2014; Frederick, 2005; Osman, 2004; Pennycook et al., 2016; Raven & Raven, 2003). As suggested above, if dual reasoning can explain the link between cognitive abilities and risk preferences, we would expect a higher correlation coefficient for studies applying the CRT. With this in mind, we categorized the administered cognitive tests in each study into one of two groups, distinguishing between standardized IQ tests (e.g., Raven’s matrices) and the CRT. For our explanatory variables’ analysis, we assigned the value 0 to standardized cognitive ability tests and the value 1 to studies applying the CRT.

#### Domain (domain)

People might show different risk-taking behavior for gains versus losses (Kuhnen, 2015; Tversky & Kahneman, 1981, 1986). Dohmen et al. (2018) argued that this might also affect the correlation between cognitive abilities and risk preferences (Dohmen et al., 2018). We therefore divided the studies into two groups: those where risk-preference-elicitation tasks included only decisions in the gain domain and those where the tasks included only decisions in the loss domain. Importantly, we did not include the mixed domain in the current analysis as tasks entailing choice options from the mixed domain measure loss aversion.

#### Type of risk-preference-elicitation task (MPL)

The level of risk taking in a choice task might also depend on whether one of the two options is a sure option or whether both options are risky gambles, leading to a phenomenon known as the certainty effect (Andreoni & Kuhn, 2019; Tversky & Kahneman, 1986). We therefore split risk-preference-elicitation methods into two groups: one for methods that incorporated options with sure outcomes, so that people had to choose between a sure option (i.e., outcomes associated with 100% probability) and a risky option (i.e., outcomes associated with probabilities <100%), and the other for methods that incorporated choices between two risky gambles (i.e., both choice options associated with probabilities <100%).

#### Number of choices (#Choices)

A longer task might reduce people’s attention. This could potentially affect people’s choice behavior such that people with higher cognitive abilities are better able to remain focused. We aimed to answer the question of whether the cognitive demands of a given risk-preference-elicitation task change the relation between cognitive abilities and risk aversion. We used the number of choices in each risk-preference-elicitation task as a proxy for the task’s cognitive demand.

### Study characteristics

In all, we examined 30 articles with a total of 18,422 participants. Nineteen studies reported correlations between multiple cognitive ability measures and risk preferences. Furthermore, seven studies assessed risk preferences by looking into multiple domains and three studies investigated the relationship between cognitive abilities and risk preferences by applying different types of price lists. Our analysis of explanatory variables shows that 58% of the studies had an RCRT bias towards risk aversion and 22.6% an RCRT bias towards risk seeking, and 16.1% had no bias (in 3.2% the classification was not possible; see Table 2). For cognitive ability tests, 63% of the studies used a standardized test (e.g., Raven & Raven, 2003) and 37% used the CRT (Frederick, 2005). Regarding the risk-preference-elicitation methods, 14.5% of the studies included choice options associated with potential losses, whereas the large majority, 85.5% of the studies, presented only gambles associated with potential gains. Also, 42% of the studies included only choices between two risky options and 58% included choices between safe and risky gambles. Lastly, 51.6% of the studies had sample sizes above 200 participants and 48.4% had smaller sample sizes (see the Appendix, Complementary Results). For the full list of the studies included in the current meta-analysis and their characteristics, see Table 2. Altogether, the collected data are organized in multiple levels, where a single study can be associated with multiple reported effects. This means that our study introduced additional levels of variability that are accounted for by a multilevel meta-analytical model with the study identifier set as a random effect.

### Statistical analysis

#### Effect-size coding

To systematically compare effect sizes, we converted standardized regression coefficients (Peterson & Brown, 2005) and values into correlation coefficients (Ellis, 2010) for the studies that did not conduct a correlation analysis and/or did not report correlation coefficients. Whenever unstandardized regression coefficients were reported, we contacted the respective authors and requested the correlation coefficient between the cognitive test administered and risk aversion (the authors of the following studies replied to our request: Booth et al., 2014; Borghans et al., 2009 Noussair et al., 2014; Taylor, 2013, 2016). For consistency, we considered only correlation coefficients as effect sizes. Following Dohmen et al. (2018), we coded all effect sizes such that a potential positive correlation between cognitive abilities and risk aversion indicates that people with high cognitive abilities show more risk-averse behavior and are classified as being more risk averse (Dohmen et al., 2018). Furthermore, we applied the Fisher *z* transformation to each correlation coefficient and computed the corresponding sampling variances of the effect sizes. Sampling variances are error terms that represent the deviance between the observed and the true effect size.

#### Statistical analysis approach

All analyses were conducted by following a Bayesian statistical approach. Specifically, we estimated multilevel linear models to evaluate the metaeffect of the link between cognitive abilities and risk aversion, while accounting for multiple levels in the data (e.g., when multiple effect sizes were reported in a single study). This approach has two main advantages: First, it allowed us to draw probabilistic conclusions about the true effect and the uncertainty around it, and second, it allowed us to utilize prior knowledge about the nature of the effect-size distribution (Wagenmakers et al., 2018). Practically, a likelihood function is applied to inform the likelihood of the observed data. For the purpose of the current analysis, we chose a Cauchy distribution to inform the mean population-level parameters (i.e., the mean and the between-study heterogeneity). The Cauchy distribution is a weakly informative prior and is characterized by a high density around its tails (Chung et al., 2015). To avoid forcing the boundaries of the prior distribution (i.e., here, because the effect sizes are correlation coefficients, the distribution’s boundaries are restricted to −1 and +1), we applied the Fisher *z* transformation to the reported correlation coefficients (i.e., causing them to lie between −∞and +∞), so that they correspond to the properties of the Cauchy probability distribution function (Fisher, 1915).

A Bayesian estimation of the metaeffect represents a joint distribution, calculated by multiplying the likelihood with the prior distribution, resulting in a posterior distribution that reflects how the prior belief is updated on the basis of the observed data. Besides the mean coefficient estimation, posterior distributions represent the uncertainty in the parameter estimate. Therefore, they can be summarized in statistics as the mean and the 95% Bayesian credible interval (95% BCI; Morey et al., 2016). The 95% BCI provides an estimate of the interval in which the true effect lies.

#### Multilevel modelling

The multilevel modelling approach is applied when the data are organized in levels that are dependent on each other. These models assume conditional dependence of the effect sizes (Pastor & Lazowski, 2018). In meta-analyses this is often the case because some studies report multiple effect sizes that stem from a single cohort. Practically, in meta-analytical models, the two types of variations are considered in two steps. First, the within-study variation is considered. In a meta-analysis, these are simply the sampling errors associated with each reported effect size. Second, the second level of variation—namely, the between-study variation—is accounted for. This is computed by estimating a metaeffect size (i.e., the mean effect across all effect sizes estimated in Level 1) and the distribution around it (i.e., the between-study heterogeneity). Thus, the inclusion of a random intercept implies that the variation within and between effect sizes is accounted for. We utilized a multilevel modelling approach to our data set as it is organized in levels. This approach is particularly crucial since the majority (63%) of the identified studies reported multiple effect sizes stemming from a single cohort. For instance, some studies applied multiple risk-preference-elicitation tasks or more than a single measure for cognitive abilities and reported an effect size for each of the applied measures. An alternative approach to random effects is the inclusion of fixed effects in meta-analytical models. In the case of a meta-analysis, the inclusion of fixed effects implies that the variation between studies will be accounted for but not the variation within a study. Consequently, if every data point (here, each study) is assumed to have a single level of variation (i.e., if a study is associated with a single reported effect size), an application of random effects is redundant since in such a case random and fixed effects will converge, and the parameters’ estimation would be identical (i.e., equal to the residual error). Importantly, in our case, it is clearly beneficial to include random effects because the majority of studies reported multiple effect sizes stemming from the same cohort, indicating potential effects of within-study variation on the metaeffect. The inclusion of fixed effects only would result in the loss of this significant information and could influence the resulting estimates (Hox, 1998). We provide a detailed analysis of both the within-study and between-study variations to provide further reasoning and support for the advantages of utilizing a multilevel meta-analytical model for our analysis (see the Appendix).

#### Meta-analytical model

We started our analysis by estimating a model to assess the overall association and associated credibility between cognitive abilities and risk aversion. We fitted a hierarchical regression model to estimate the random intercept with correlation coefficients as a dependent variable (𝛒 = correlation coefficients_Z ― transformed)_, 𝛃_𝟎_ = intercept) (c.f.*M*_*n*_) (see Equation 1). Next, we examined the importance of each explanatory variable in explaining the relation between cognitive abilities and risk aversion. Therefore, we estimated a regression model that included all possible variables (see Equation 2). Importantly, we investigated further simplified versions of this model (see Table 5).

**Table 5:** Linear models examined for explanatory variable selection procedure applying hierarchical regression procedure

Explanatory variable	*M*_*n*_	*M*_*f*_	*M*_*1*_	*M*_*2*_	*M*_*3*_	*M*_*4*_	*M*_*5*_
Intercept	−**.03** [−.08, .02]	**.11** [.03, .19]	−**.02** [−.12, .07]	**.11** [.03, .19]	**.13** [.04, .20]	**.11** [.05, .17]	**.11** [.03, .19]
RCRT bias (proportion of risk-averse options)	—	−**.26** [−.31, −.21]	—	−**.26** [−.31, −.21]	−**.28** [−.32, −.23]	−**.26** [−.30, −.21]	−**.26** [−.31, −.21]
Type of cognitive test (CRT applied)	—	−**.003** [−.04, .03]	**-.01** [−.05, .03]	—	−**.003** [−.04, .03]	−**.002** [−.04, .03]	−**.003** [−.04, .03]
Domain (potential loss included)	—	**.02** [−.02, .07]	**.16** [.12, .19]	**.02** [−.02, .07]	—	**.02** [−.02, .06]	**.02** [−.02, .06]
Type of choice options (sure-payment option included)	—	−**.03** [−.12, .06]	**.01** [−.10, .12]	**-.03** [−.12, .07]	**-.03** [−.12, .06]	—	**-.03** [−.12, .06]
Number of choice options	—	**-.0002** [−.003, .003]	**-.003** [−.006, .0004]	**-.0002** [−.003, .003]	**-.00027** [−.0003, .003]	**-.0006** [−.003, .002]	—
BF	**4.3 × 10**^**31**^	1	**1.0 × 10**^**21**^	**.045**	**.10**	**.05**	**.003**

*M*_*n*_*–M*_*5*_ are linear regression models. *M*_*n*_ is a random-intercept model. *M*_*f*_ is a full model that includes all recorded explanatory variables. The estimated posterior means are indicated in bold font. The 95% Bayesian credible intervals are indicated in brackets. Dependent variables are correlation coefficients and the corresponding sampling errors. The explanatory variables are RCRT bias—a continuous explanatory variable for assessing risk-taking propensity in the case of random error, type of cognitive test—a dummy-coded explanatory variable for differentiating between standardized IQ tests and other tests for assessing cognitive abilities, domain—a categorical explanatory variable representing domain with two levels (gain domain, loss domain), type of choice options—a dummy-coded variable for differentiating between elicitation methods consisting of a safe and a risky option and elicitation methods consisting of two risky options, and number of choice options—a continuous variable representing the number of choices included in the elicitation methods. BF refers to the Bayes factor applied to compare between a full model and the respective model. CRT = Cognitive Reflection Test; RCRT = random choice risk taking

1 $${\mathrm{P}}_{ij}={\upbeta}_0+{\mu}_j+{\epsilon}_{ij},$$

2 $${\uprho}_{ij}={\upbeta}_0+{\upbeta}_1\bullet {D}_{\mathrm{RCRT}}+{\upbeta}_2\bullet {D}_{\mathrm{CAT}}+{\upbeta}_3\bullet {D}_{\mathrm{MPL}}+{\upbeta}_4\bullet {\mathrm{MPL}}_{\#\mathrm{Choices}}+{\upbeta}_5\bullet {D}_{\mathrm{Domain}}+{\mu}_j+{\epsilon}_{ij},$$

where *i* represents the reported effects sizes and *j* represents the effects sizes nested within each study. Hence, in Equation 1, ρ_*ij*_ represents the dependent variable, β_0_ is the fixed predictor, *μ*_*j*_ represents the random intercept drawn from a Cauchy distribution and *ϵ*_*ij*_ represents the residual variance.

#### Statistical analysis software

All analyses were conducted using the statistical program R (Version 3.2.1; R Core Team, 2014). The joint posterior parameter distributions were estimated using Monte Carlo Markov Chain methods implemented in the brms package, an R interface to Stan (Bürkner, 2017a, 2017b). We ran four parallel chains for 10,000 iterations each. Three thousand additional samples were used as a warm-up for each of the chains and were therefore discarded. In addition to visually inspecting the plots of each chain, we conducted Gelman–Rubin statistical diagnostics, which provides information on the convergence of the algorithm (Gelman & Rubin, 1992).

#### Heterogeneity

To consider the sources of heterogeneity in our sample we applied both Cochran’s *Q* and *I*^2^ statistics. The *I*^2^ statistic describes the percentage of variation across studies that is due to heterogeneity rather than chance and is advantageous since it does not inherently depend on the number of studies considered (Higgins et al., 2003; Higgins & Thompson, 2002). In other words, *I*^2^ is a quantified expression of the inconsistency of studies’ results. This helps identify the source of differences between effect sizes using identified potential moderators (see Table 4).

#### Influential outliers

**Fig. 2:**
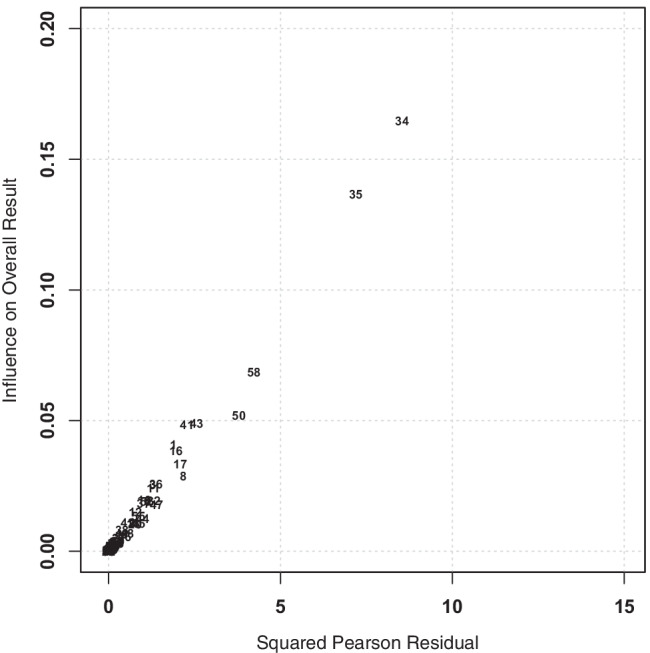
Baujat plot of the correlation coefficients between risk aversion and cognitive abilities (denoted by the first study identifier) plotted according to their influence. *Note.* The *x*-axis represents the contribution of each effect size to the overall heterogeneity. The *y*-axis represents the influence each study has on the overall result. The most heterogeneous and influential studies appear in the upper-right area

We detected potential outliers in two ways. First, we visualized the studies’ contribution to the heterogeneity using a Baujat plot (Baujat et al., 2002). The Baujat plot uses the *Q*-test statistic for heterogeneity on the *x* axis and the influence of each study on the *y*-axis (see Fig. 2). Heterogeneity is defined as the standardized squared difference between the overall estimate based on a fixed-effects model with the study included in the model fitting and the overall estimate based on a fixed-effects model without that inclusion. Second, to analytically pinpoint potential outliers, we computed Cook’s distances for all effect sizes (see Fig. 10 in the Appendix) and ran a sensitivity test to learn about the influence of the identified outliers.

#### Publication bias

**Fig. 3:**
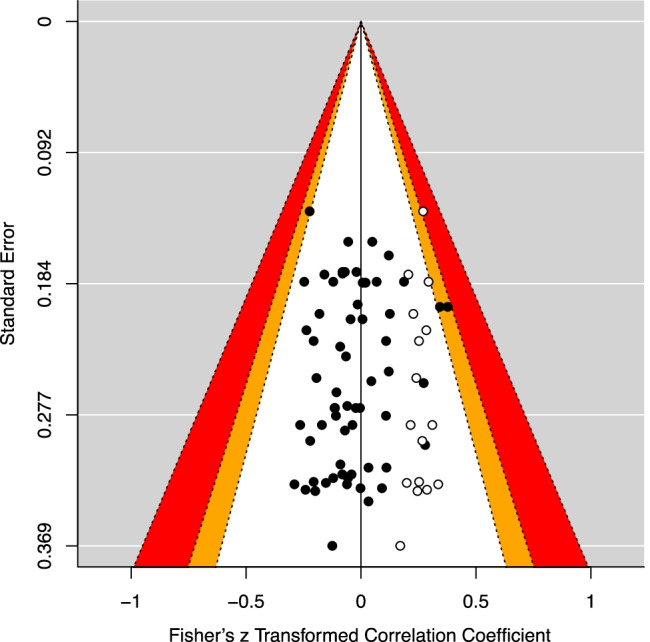
Funnel plot with standard error for effect sizes (i.e., correlation coefficients) of the link between risk aversion and cognitive abilities. *Note.* The red zone represents the area of statistical significance (effect sizes between .1 and .01). Effect sizes (i.e., correlation coefficients) lay within both the negative and the positive regions and mostly within the nonsignificance zone, creating a relatively symmetrical funnel plot (i.e., no indication of a publication bias)

Publication bias is a known phenomenon in academic research that occurs when the outcome of a research study influences the decision of whether to publish it. Significant results are more likely to be published (Rothstein et al., 2004). To uncover potential publication bias, we first created a funnel plot, a useful tool to visualize studies’ effects plotted against their standard errors, a determinant of precision (see Fig. 3). Second, we used the trim-and-fill method to lay ‘absent’ studies necessary to achieve funnel plot asymmetry (Duval & Tweedie, 2000). We tested the funnel plots symmetry with Egger’s regression test, a linear regression test of each effect size against its associated estimated precision (Egger et al., 1997).

#### Explanatory variable selection

To test the robustness of the explanatory variable’s mean estimates against the presence of other explanatory variables, we used and defined five other models (see Equation 2). We excluded one explanatory variable in each additional model, leaving all others unchanged (see Table 5). We ran the robustness check by using an approach for the model assessment, in which the aim is to test how the exclusion of an explanatory variable improves the model fit compared with a full model (see Equation 2). We estimated five models, excluding variables one at a time. Each explanatory variable’s importance was determined by its slope mean posterior estimation and its associated 95% BCI. Moreover, we compared the defined models using Bayes factors (BFs). BFs are used to compute the posterior odds for one model (e.g., *M*_*a*_) against a second model (e.g., *M*_*b*_) by updating the prior odds with the diagnostic information from the data (Shiffrin et al., 2008; see Table 5). Hence, a higher value suggests the superiority of *M*_*a*_ over *M*_*b*_. Here, the prior odds of the full model (i.e., *M*_*f*_) are tested in relation to the prior odds of each of the defined models (i.e., *M*_*1-5*_; see Table 5).

We examined the importance of each explanatory variable with a model comparison procedure. That is, we compared a model that discards the explanatory variable of interest to a full model that includes all explanatory variables (see Equation 2 and see Table 5). This way, we determined which of the two compared models better fits the data. Table 5 shows the results.

#### Data availability

Both the analysis scripts and the data are available (https://osf.io/m4qz9/).

## Results

We first report the results from the meta-analytical model, which shows a substantial degree of heterogeneity among the identified studies. Subsequently, we report the exploratory analysis investigating the sources of this heterogeneity.

### Noncredible metaeffect size

We estimated the size of the metaeffect of the link between cognitive abilities and risk aversion by estimating a random-intercept multilevel linear model with clustering for study identifier on the study level (see Equation 1). We determined the metaeffect and its credibility by inspecting the intercept’s mean estimate and the 95% BCI around it. Model in Table 5 reports a mean estimate of −.03 with a 95% BCI ranging from −.08 to .02. The results reveal a negative, negligible metaeffect of the link between cognitive abilities and risk aversion. Therefore, we conclude that across all studies it is probable that *no* association between cognitive abilities and people’s risk preferences exists, speaking against the risk-preference hypothesis.

### Publication bias

We looked for potential publication bias—that is, that only significant results were published (see Rothstein et al., 2004). The Egger’s regression test does not indicate an asymmetrical distribution of the effect sizes (*z* = −.23, *p* =.82), represented by the funnel plot in Fig. 3. Thus, we conclude that the data show no publication bias and that our data consist of equally distributed significant and nonsignificant effect sizes.

### Heterogeneity across studies

**Fig. 4:**
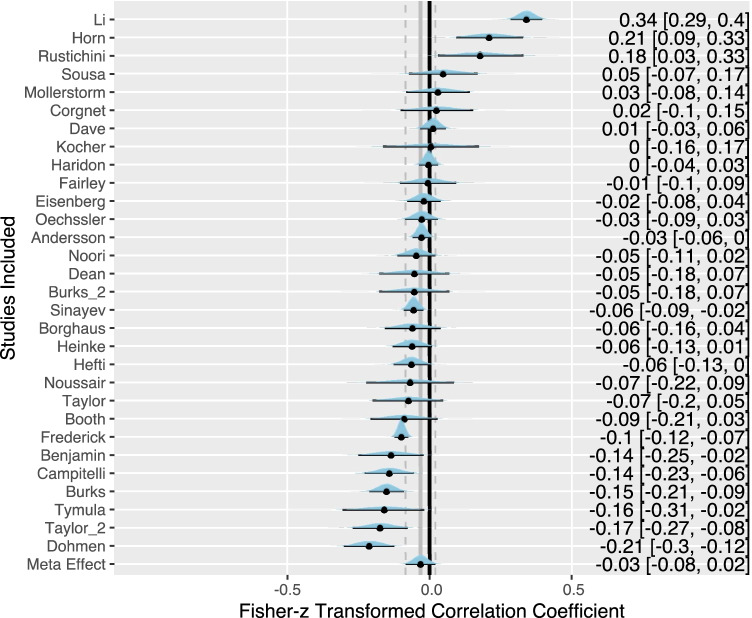
Forest plot with posterior distributions for each study. *Note.* The meta-analytic posterior distribution is displayed on the bottom row. The *y* axis shows the names of first authors of all included studies. The graph shows the mean posterior estimates (black filled points and values on the right) and the associated 95% Bayesian credible intervals (the values in squared brackets) against the zero point (grey solid line). For most studies a negative value can be observed (with 11 studies showing a credible negative value). This implies a negative correlation between cognitive abilities and risk aversion, and thus people with low cognitive abilities show more risk aversion. Positive values indicate a positive correlation between cognitive abilities and risk aversion, so that people with low cognitive abilities show less risk aversion. For six studies a credible positive value was estimated. The Fisher *z* transformation is a normalizing transformation for correlation coefficients. Fisher *z* values of −1.0 and +1.0 correspond to correlation coefficients of approximately .8 and −8, respectively

Figure 4 summarizes in a forest plot all study posterior distributions ordered by the magnitude of their estimated effect sizes and estimated intercepts. While the meta-analytical model reveals an overall noncredible effect size for the relationship between cognitive abilities and risk aversion, Fig. 4 shows some heterogeneity across studies. Using the Cochran *Q* test, we see significant variation among the collected effect sizes with *Q*(48) = 692.16, *p* < .0001. Furthermore, the *I*^2^ index indicates a substantial proportion of heterogeneity among reported effect sizes with *I*^2^ = .90. Thus, 90% of the effect sizes’ variation cannot be explained by one common cause.

### Explanatory variable analysis

The Cochran *Q* test and the *I*^2^ test results indicate that substantial variation among effect sizes exists. We conducted an exploratory analysis by testing potential study characteristics (see Method, Study Coding section) and tested their explanatory power to explain the observed heterogeneity.

#### RCRT bias (RCRT)

Following the error hypothesis, the presented choice set in a particular risk-preference-elicitation task may lead to the misclassification of a decision maker with random choices as risk averse or risk seeking. To facilitate an understanding of the differences among the RCRT-classified groups (i.e., RCRT-bias towards risk seeking, RCRT-bias towards risk aversion, and no RCRT bias), we calculated the raw mean effect size for those studies that applied a task architecture that fell under the RCRT categorization as (a) biased towards risk seeking: .01 (*SD* = .14) with a total of *n* = 14 reported correlations, (b) biased towards risk aversion: −.10 (*SD* = .10) with a total of *n* = 36 reported correlations, and (c) no bias: .02 (*SD* = .12) with a total of *n* = 10 reported correlations. Thus, studies with an RCRT bias towards risk aversion reported a stronger negative correlation between cognitive abilities and risk aversion. Table 5 presents the role of the task architecture variables in the reported correlation between cognitive abilities and risk aversion.

**Fig. 5:**
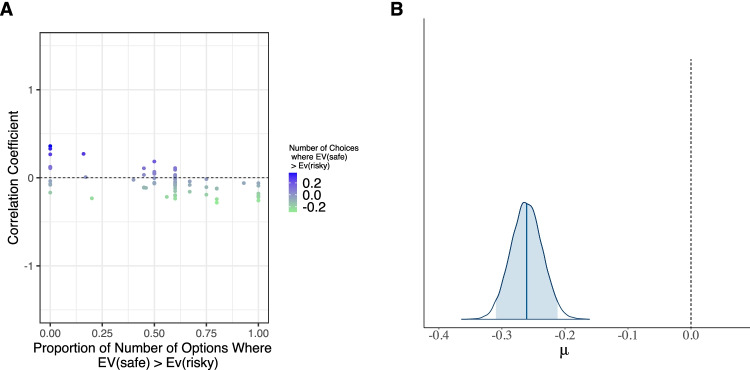
Raw data of the RCRT-bias variable and its corresponding estimated posterior distribution. *Note.* Panel **a**: Raw data of the proportion of number of options in which the expected value (EV) of the less risky choice options is larger than the EV of the riskier ones, plotted against the corresponding correlation coefficients of the link between risk aversion and cognitive abilities. Panel **b**: The estimated posterior mean of the RCRT-bias variable (i.e., the proportion of choices in which the EV of the less risky choice options is larger than the EV of the riskier ones). Note that the estimated posterior mean, indicated by a solid blue line in the middle of the distribution, differs substantially from the zero point (indicated by the grey dotted line). The shaded area of the posterior distribution of the RCRT bias represents the 95% Bayesian credible interval. RCRT = Random choice risk taking

Turning to the effect of an RCRT bias on the reported correlation, the results in Table 5 (see Models *M*_*f*_*, M*_*2*_*–M*_*5*_) show that across all specifications, the proportion of options where the EV of the safe option was greater than the EV of the risky option (RCRT bias towards risk aversion, see Method) exhibits a credibly negative mean estimate of −.26 and a 95% BCI ranging from −.31 to −.21 (see Fig. 5b). This result reveals that the larger the fraction of risk-averse decisions (i.e., the larger the number of items where the EV of the risky option is larger than the EV of the safe one) in a risk-preference-elicitation task, the lower will be the observed correlation between cognitive abilities and risk taking. This finding is in line with the error hypothesis. That is, people with lower cognitive abilities make more errors and tend to make choices that move towards random choice behavior. If a task is constructed such that random choices are interpreted as a specific risk preference, a correlation between cognitive abilities and risk preferences could be spurious and misinterpreted.

As for the relative importance of this variable (compared with other moderators), the BF comparing the full model that includes the RCRT bias (see Table 5, Model *M*_*f*_) with a model that discards it (see Table 5, Model *M*_*1*_) exhibits the overwhelming superiority of the full model. Overall, in line with previous claims (e.g., Andersson et al., 2016), our results support the interpretation that a risk-preference-elicitation method that is biased toward risk aversion imposes an effect on the reported correlation between cognitive abilities and risk aversion.

#### Cognitive Ability Test (CAT)

**Fig. 6:**
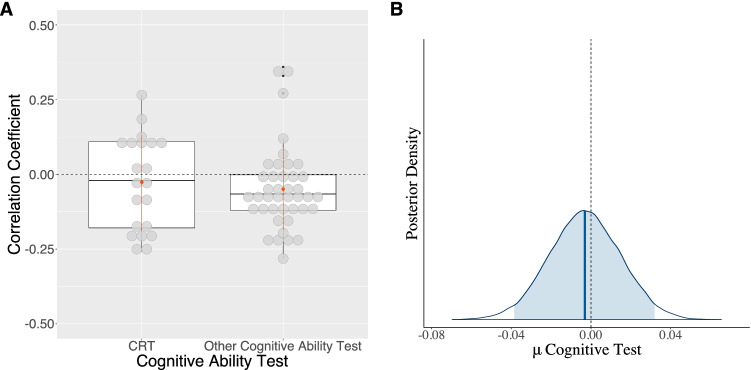
Raw data of the CRT variable and its corresponding estimated posterior distribution. *Note.* Panel **a**: Raw data of the applied cognitive tasks associated with each study and the mean difference of the correlation coefficients of the link between risk aversion and cognitive abilities against the zero point (black dashed line). CRT represents the studies that used the Cognitive Reflection Test to measure cognitive abilities. Other Cognitive Ability Test refers to all other types of cognitive ability tests (e.g., Raven’s matrices). Panel **b**: The estimated posterior mean of the difference between the two categories of cognitive tests. Note that the estimated posterior mean, indicated by a solid blue line in the middle of the distribution, does not differ from the zero point (indicated by the grey dotted line). The shaded area of the posterior distribution of the cognitive test represents the 95% Bayesian credible interval

As suggested in the Introduction, dual reasoning could explain a potential link between cognitive abilities and risk aversion. Accordingly, one would expect larger correlation coefficients for studies applying the CRT, as this test is meant to distinguish between intuitive and deliberate reasoning styles (see Frederick, 2005, for a more detailed explanation). The results in Table 5 (Models *M*_*f*_ *– M*_*5*_) show that across all specifications, studies using the CRT do not differ from those using standardized cognitive ability tests. This is demonstrated by the noncredible mean estimate for the dummy variable CRT as a cognitive test with a mean estimate of −.003 and a 95% BCI ranging from −.04 to .03 (see Table 5, Model *M*_*f*_ and Fig. 6b). Similarly, the model comparison favours the model that discards this variable compared with a model that includes it (see Table 5, Models *M*_*f*_*, M*_*2*_). Thus, measures that aim to distinguish different reasoning styles cannot explain the heterogeneity in the effect sizes.

#### Domain (domain)

**Fig. 7:**
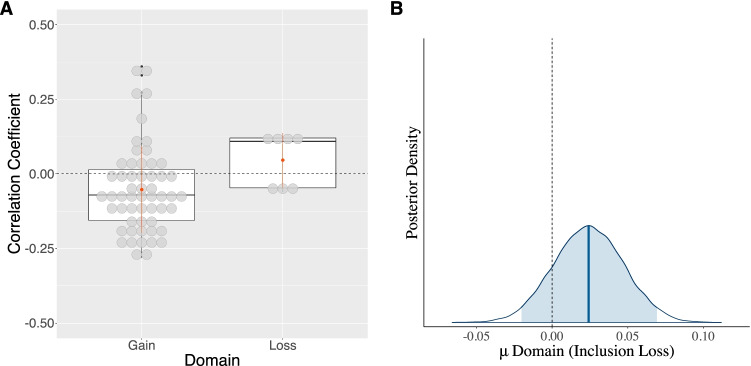
Raw data of the domain variable and its corresponding estimated posterior distribution. *Note.* Panel **a**: Raw data of the domain associated with each study and the mean difference of the correlation coefficients of the link between risk aversion and cognitive abilities against the zero point (black dashed line). Gain and Loss refer to the studies that elicited risk preferences with tasks that incorporated only potential gains or included potential losses, respectively. Panel **b**: The estimated posterior mean of the difference between the two domains. Note that the estimated posterior mean, indicated by a solid blue line in the middle of the distribution, differs from the zero point (indicated by the grey dotted line). The shaded area of the posterior distribution of the domain represents the 95% Bayesian credible interval

Following Dohmen et al. (2018), we examined whether task framing influences the direction of the correlation between cognitive abilities and risk aversion (Dohmen et al., 2018). Studies using tasks including losses had an average raw effect size of .04 (*SD* = .09) compared with −.05 (*SD* = .14) for studies using tasks including only gains. The effect of domain on the link between risk aversion and cognitive abilities is apparent by the mean posterior estimate of .02 and a 95% BCI ranging from −.02 to .07 (see Table 5, Model *M*_*f*_ and Fig. 7b). The BF comparison of the full model, *M*_*f*_, provides anecdotal evidence of the superiority of including the explanatory variable domain compared with a model that excludes it (see Table 5, Models *M*_*f*_*, M*_*3*_).

Across the models’ specifications, RCRT bias changes as a result of the inclusion of the domain. Hence, we examined the correlation between domain and an RCRT bias (see Appendix Table 7). The results suggest that the two variables relate to one another. To determine whether the domain or the RCRT bias should be included in our meta-analytical model, we compared a model that includes all explanatory variables (with the RCRT*-*bias variable) *except for* the domain variable (see Table 5, Model *M*_*3*_) with a model that includes all explanatory variables (with the domain variable) *except for* the RCRT-bias variable (see Table 5, Model *M*_*1*_). This comparison exhibits strong evidence in favour of the model that excludes the domain variable and includes RCRT-bias over a model that excludes the RCRT-bias and includes the domain variable (*BF*_*M3, M1*_ = 9.8×10^21^). Overall, these findings provide further evidence that random error strongly affects the correlation between cognitive abilities and risk aversion.

#### Type of risk-preference-elicitation task (MPL)

**Fig. 8:**
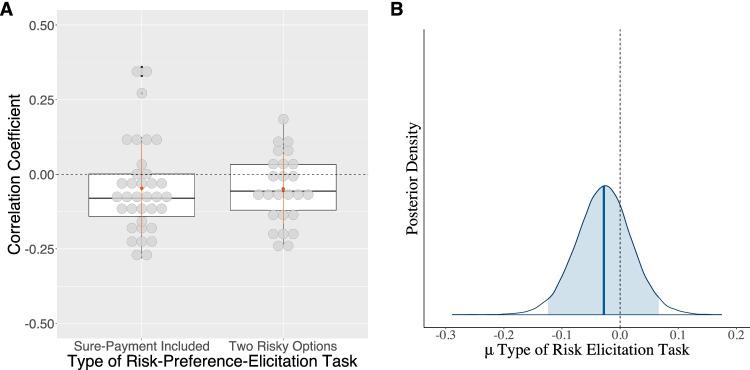
Raw data of the type of risk-elicitation task variable and its corresponding estimated posterior distribution. *Note.* Panel **a**: Raw data of the type of the risk preference elicitation task associated with each study and the mean difference of the correlation coefficients of the link between risk aversion and cognitive abilities against the zero point (black dashed line). Sure-payment included represents studies that elicited risk preferences with tasks that involved a sure-payment option. Two risky options refers to tasks that involved a choice between two risky options. Panel **b**: The estimated posterior mean of the difference between the two types of risk elicitation task. Note that the estimated posterior mean, indicated by a solid blue line in the middle of the distribution, does not differ from the zero point (indicated by the grey dotted line). The shaded area of the posterior distribution of the type of the risk preference elicitation task represents the 95% Bayesian credible interval

To test whether the certainty effect moderates the correlation between cognitive abilities and risk aversion, we included the explanatory variable in our meta-analytical model. Our results show that across all specifications (see Table 5, Models *M*_*f*_*–M*_*5*_), risk-preference-elicitation methods that include a sure-payment option do not differ from those that include only risky choice options. Consistently, the noncredible estimate for the sure-payment-included variable has a mean of −.03 and a 95% BCI ranging from −.12 to .06 (see Table 5, Model *M*_*f*_*,* also see Fig. 8b). The model comparison shows that the model that discards this variable is better than the model that includes it (see Table 5, Model *M*_*4*_). Hence, inclusion or exclusion of sure options does not substantially affect the correlation between cognitive abilities and risk preferences.

#### Number of choice options (#Choices)

**Fig. 9:**
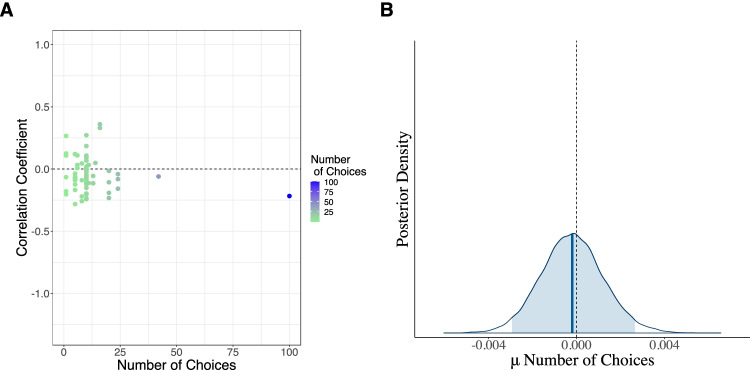
Raw data of the number of choice options variable and its corresponding estimated posterior distribution. *Note.* Panel **a**: Raw data of the number of choice options associated with each risk elicitation task for each of the reported studies plotted against the corresponding correlation of the link between risk aversion and cognitive abilities against the zero point (black dashed line). Number of choices refers to the number of choices in the risk-preference-elicitation tasks. Panel **b**: The estimated posterior mean of the difference between the number of choices. Note that the estimated posterior mean, indicated by a solid blue line in the middle of the distribution, does not differ from the zero point (indicated by the grey dotted line). The shaded area of the posterior distribution of the number of choices represents the 95% Bayesian credible interval

**Fig. 10:**
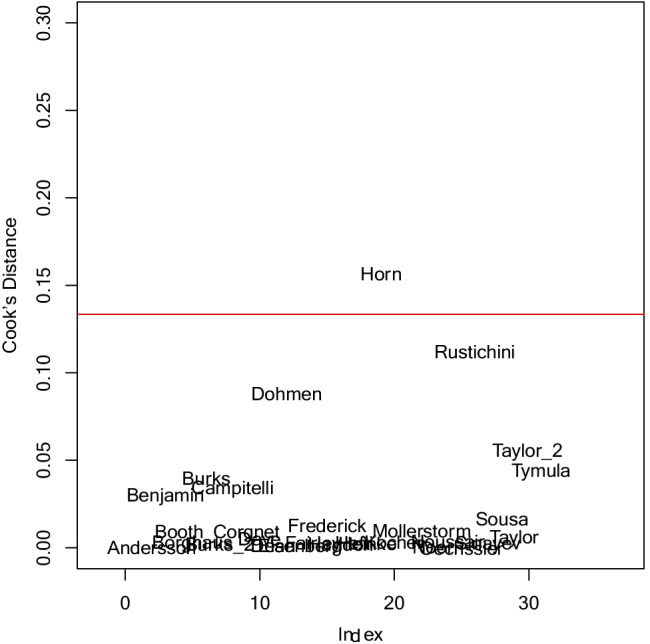
Cook’s distance plot. *Note.* The names represent the Cook’s distance computed for each reported effect size. The red horizontal line represents the cut-off line

We tested whether the length of a risk-preference-elicitation task moderates the correlation between cognitive abilities and risk aversion. Our full model exhibits a mean estimate for the number of choice options variable of −.0002 with a 95% BCI ranging from −.003 to .003 (see Fig. 9b and Table 5, Models *M*_*f*_*–M*_*5*_). A model that neglects this explanatory variable (see Table 5, *M*_*5*_) is preferred over a model that includes it (see Table 5, Model *M*_*f*_). Thus, the length of the risk-preference-elicitation task does not affect the correlation between cognitive abilities and people’s risk preferences.

Altogether, our results reveal the striking importance of the risk-preference-elicitation task’s architecture, in particular its sensitivity to an RCRT bias towards risk aversion, in determining the direction and magnitude of the link between cognitive abilities and risk aversion. This effect is shown by a robust, credible, and strong negative mean coefficient estimation of the moderator variable representing the RCRT bias. Thus, a negative correlation between cognitive abilities and risk aversion is larger in those studies that applied risk-preference-elicitation tasks that were affected by random choice behavior. If the task architecture promotes an RCRT bias, the chance of finding a correlation between cognitive abilities and risk preferences is enhanced. In sum, our results provide evidence that no systematic link between cognitive abilities and risk aversion exists. We further find an effect of random choice behavior that can be misinterpreted as a specific risk preference on the reported correlation between risk aversion and cognitive abilities. Furthermore, our results suggest that all other characteristics had no moderating effect.

## Discussion

We conducted a Bayesian meta-analysis with a total of 30 studies and examined whether a potential meta effect size is better explained by the risk-preference hypothesis, which assumes a correlation between cognitive abilities and risk aversion because cognitive abilities affect the evaluation of risky options and, consequently risk-taking behavior, or by the error hypothesis, which assumes that mixed results are the product of a relationship between cognitive abilities and decision errors resulting from a bias of the architectural properties of the risk-preference-elicitation task. Our results show that the correlation between cognitive ability and risk aversion is noncredible. Notably, we find that when studies applied unbalanced choice sets, they reported a stronger negative (or positive) correlation between cognitive abilities and risk aversion depending on the direction of this unbalance. The effect of the RCRT bias was robust across all meta-analytical model specifications and thus provides strong evidence for the error hypothesis. That is, our findings support the claim that previous mixed evidence of a correlation between cognitive abilities and risk aversion is mainly driven by the important interaction between the architecture of the risk-preference-elicitation task and errors in decision making. In addition, we found an effect of task framing, where including losses in risk-preference-elicitation tasks only weakly moderates the relation between cognitive abilities and risk aversion. Note that this effect was not robust across all meta-analytical model specifications and appears to be highly correlated with the RCRT bias of the choice set, where the latter has a higher explanatory power. We found no mediating effects of the type of cognitive ability test applied or of the number of decisions. We conclude that a potential correlation between cognitive abilities and risk aversion is moderated by the link between cognitive abilities and the probability of making unsystematic decision errors.

A recent meta-analysis by Lilleholt (2019) similarly explored the link between cognitive abilities and risk preferences. However, in contrast to our work, Lilleholt’s analysis did not directly test whether the mixed findings regarding the link between cognitive abilities and risk preferences could be explained by the error hypothesis and the bias in the architecture of most risk-preference-elicitation tasks. There are other important differences. First, Lilleholt had a broader literature search scope, leading to a larger set of examined studies. For instance, the author included experience-based risk-preference-elicitation tasks, which we excluded from our analysis. In such tasks, people have no information about the outcomes of gambles and the probabilities with which the outcomes occur but learn this from feedback. Thus, in these tasks, learning plays a major role in how people make their decisions, thereby making the interpretation of a potential link between cognitive abilities and risk preferences more complicated. In general, it has been argued that description-based and experience-based tasks differ in both architecture and interpretation (Frey et al., 2017). Therefore, in contrast to Lilleholt, we have focused on a description-based task that makes it easier to code all relevant task-architecture information precisely.

Since Lilleholt (2019) ran the meta-analysis for each domain separately, we compared Lilleholt’s results with our results by estimating our meta-analytic models on Lilleholt’s merged data set (see Appendix Table 8). In line with Lilleholt’s results, we find a credible metaeffect of −.05 with a 95% BCI ranging from −.07 to −.03 for the loss, gain, and mixed domains. Note that our restricted data set exhibits a comparable effect size of −.03, with a 95% BCI ranging from −.08 to .02. Additionally, the inclusion of losses as outcomes of the choice options had a credible effect on the correlation between cognitive abilities and risk preferences with a mean estimate of .12 and a 95% BCI ranging from .08 to .15 (see Appendix Table 8, Model *M*_*f*_). The model comparison shows that the model that includes this variable is superior to a model that exclude it (see Appendix Table 8, Models *M*_*f*_*, M*_*2*_). The effect of the RCRT bias towards risk aversion on the correlation between cognitive abilities and risk preferences was credible across all model specifications (see Appendix Table 8, Models *M*_*f*_*, M*_*1,*_ *M*_*2*_) and exhibited a mean estimate of −.17 and a 95% BCI ranging from −.25 to −.09 (see Appendix Table 8, Model *M*_*f*_). More importantly, a regression model comparison procedure (see Appendix Table 8) shows that accounting for RCRT bias (*M*_*f*_ vs*. M*_*1*_ *BF* = 5.9×10^6^) and the inclusion of losses (*M*_*f*_ vs*. M*_*2*_, *BF* = 2.1×10^10^) improve the model fit substantially for the merged data set of Lilleholt (2019), replicating our results. However, given the larger set of studies in Lilleholt compared with ours, this replication should be interpreted with caution.

Our finding of a moderating effect of an RCRT-biased task architecture on the correlation between cognitive ability and risk aversion contributes to the discussion in the decision sciences and experimental economics literature. For instance, in line with the error hypothesis, Andersson et al. (2016) experimentally demonstrated that the link between cognitive abilities and risk aversion is spurious, as it is moderated by the link between cognitive abilities and random choice behavior (Andersson et al., 2016). In keeping with this result, Olschewski et al. (2018) reported that in risk-taking tasks, cognitive abilities correlated negatively with decision errors. We followed this work and rigorously tested the error hypothesis with a meta-analysis. Our results show that the correlation between cognitive abilities and risk aversion can be explained by the characteristics of the choice set (i.e., task architecture), implying an RCRT bias, a phenomenon that leads to misclassifying random choices as a specific risk preference.

Our findings support the view of the error hypothesis that cognitive abilities are linked to the probability of making unsystematic errors (Burks et al., 2008; Dean & Ortoleva, 2015; Olschewski et al., 2018; Tymula et al., 2013). Additionally, it is plausible to assume that people with lower cognitive abilities apply simpler decision strategies (i.e., heuristics) that reduce information-processing load. However, the use of heuristics does not necessarily imply more or less risk-taking behavior; only the interaction between the applied heuristic and the task architecture leads to a specific observed risk-taking behavior. As we discussed above, some heuristics lead to higher (or lower) observed risk-seeking behavior compared with more complex decision strategies, depending on the choice set. Therefore, one would not necessarily expect a specific correlation between people’s cognitive abilities and the observed risk-taking behavior across the different tasks, but instead expect some heterogeneity in the results. However, the use of specific strategies cannot explain the relationship between the observed average risk preferences in a task and the RCRT bias in the task. Thus, the link between cognitive abilities and the selected decision strategies does not imply a link between cognitive abilities and the latent risk preferences. Crucially, when examining the potential link between cognitive abilities, decision strategies, and risk preferences, it is necessary to first identify the specific strategies people apply in specific environments or task architectures (Olschewski & Rieskamp, 2021; Rieskamp, 2008; Rieskamp & Hoffrage, 1999, 2008; Rieskamp & Otto, 2006). Future work should examine the different heuristics and decision strategies to arrive at a comprehensive understanding of whether and how those shape the correlation between cognitive abilities and risk preferences.

The results of this study also resonate with a recent empirical discourse on the validity of risk-preference-elicitation measures. For instance, Frey et al. (2017) and Pedroni et al. (2017) found behavioral risk-elicitation tasks to be less stable elicitations of risk preferences compared with self-reported measures. Importantly, the difference between behavioral and self-reported measures could disappear once measurement errors are accounted for (Andreoni & Kuhn, 2019) by applying better task architectures.

Our results also have implications for interpreting experimental results in other research domains. For example, when testing for a specific treatment effect it appears important to control for increased decision errors, so that a potential increase in errors is not misinterpreted as a specific treatment effect. Whether such misinterpretation is likely to occur depends on whether the task architecture has a bias, so that random choice behavior leads to a specific psychological interpretation. For instance, a potential effect of increased time pressure on people’s risk preferences could also simply be due to an increase in decision errors under high time pressure (e.g., Olschewski & Rieskamp, 2021). Likewise, the potential effect of cognitive load on people’s risk preferences, intertemporal time preferences, or social preferences could also simply be due to an increase in decision errors under cognitive load manipulations (e.g., Olschewski et al., 2018). Finally, the potential effect of increased monetary incentives on people’s preferences could also be due to lower decision errors with higher monetary incentives (e.g., Holt & Laury, 2002; Smith & Walker, 1993). In general, treatment effects on preferences have been observed in intertemporal discounting (e.g., Deck & Jahedi, 2015; Ebert, 2001; Hinson et al., 2003; Joireman et al., 2008) as well as social preferences (e.g., Cappelletti et al., 2011; Halali et al., 2014; Schulz et al., 2014). Across these domains, it is important to understand how changes in decision errors affect preference measurements. Failure to do so could potentially lead to misinterpretations of observed effects.

Consequently, addressing the issue of decision errors captured by the error hypothesis is of general importance to any research in behavioral economics and psychology with the objective to elicit individual preferences. There are two possible ways to address this matter. First, one can account for random errors ex ante by choosing an experimental design that controls for random errors. At the experimental design stage, researchers could apply a variety of measures to assess people’s preferences. In this way, they could cancel out systematic errors and minimize measurement errors in the associated biased classifications (Frey et al., 2017). For instance, Andersson et al. (2016) suggested choosing a symmetrical choice set when measuring risk preferences. However, this approach may not always be suitable for every preference-elicitation task. Leading to the second approach, one can account for *error* at the data analysis stage. For example, accounting for potential biases with an explicit structural decision-making model what includes an error theory at the data-analysis stage could be advantageous (Andersson et al., 2020). Recently, behavioral economists Gillen et al. (2015) and Andreoni and Kuhn (2019) proposed an instrumental variable approach to address this problem (see also Gillen et al., 2015).

It is important to note the task architecture determines the context in which a choice option is presented. Consequently, various theories relating to the context effect could also contribute to the fact that people with lower cognitive abilities are more prone to be influenced by the task architecture. For example, Andraszewicz and Rieskamp (2014) and Andraszewicz et al. (2015) demonstrated that pairs of gambles with the same differences in expected values and the same variances (i.e., risk) but various covariances (i.e., similarity) result in more unsystematic choices when the covariance between the two gambles is lower (Andraszewicz et al., 2015; Andraszewicz & Rieskamp, 2014). This effect called the covariance effect results from the fact that pairs of gambles with low covariances are more difficult to be compared with each other. Simonson and Tversky (1992) demonstrated that context effects can result from the available sample of choice options, such that extreme outcomes may appear as extreme in face of the available sample (Simonson & Tversky, 1992). Along the same lines, Ungemacht et al. (2011) demonstrated that people’s preferential choices depend on one’s exposure to hypothetical choice options.

To summarize, this meta-analysis highlights the importance of accounting for choice-set architecture, in particular, its interaction with random decision errors. Our applied methods and results go beyond the current research scope and suggest that neglecting the effect of random decision errors at the experimental design stage or at the data-analysis stage can lead to spurious correlations and the identification of “apparently new” phenomena (Gillen et al., 2019). The findings presented in this meta-analysis offer an important contribution to the scientific communities in judgment and decision making, psychology, experimental finance, and economics. In these fields of studies, measuring risk-taking propensity is particularly important. Therefore, findings of the current meta-analysis are very relevant to all researchers investigating risk-taking behavior using common risk-preference-elicitation methods.

## Figures and Tables

**Fig. 11:**
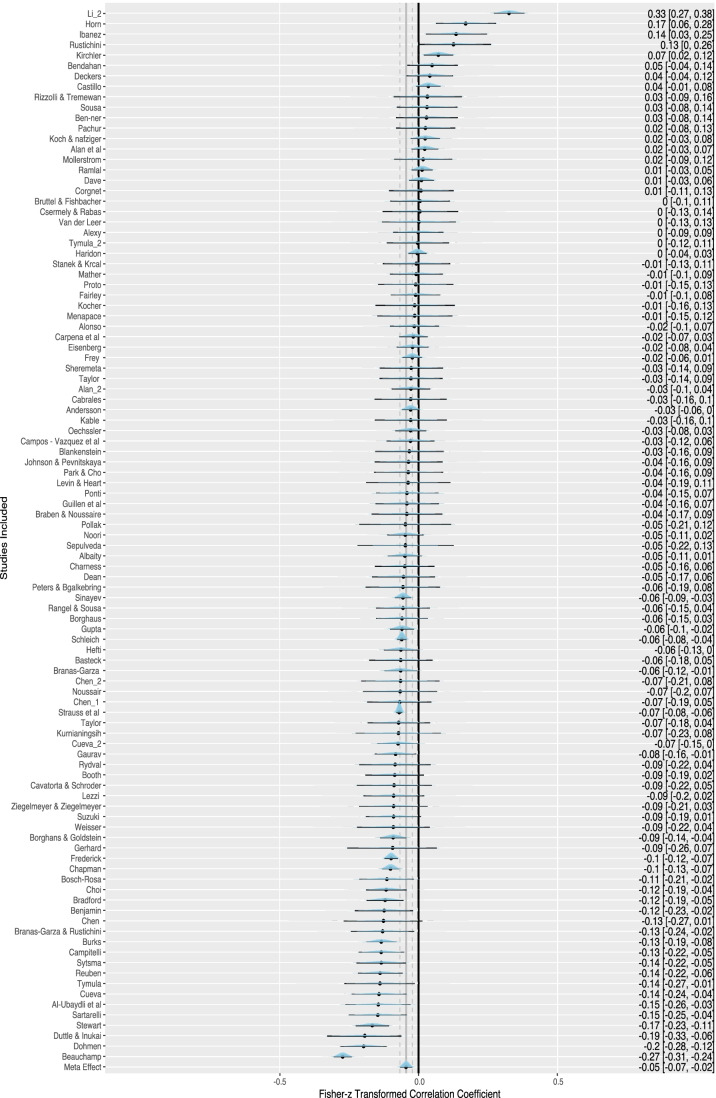
Forest plot of the effect sizes ordered by magnitude estimated for Lilleholt (2019). *Note.* The meta-analytic posterior distribution is displayed on the bottom row. The *y-*axis shows the names of first authors of all included studies. The graph shows the mean posterior estimates (black filled points and values on the right) and the associated 95% Bayesian credible intervals (the values in squared brackets) against the zero point (grey solid line). For most studies a negative value can be observed. This implies a negative correlation between cognitive abilities and risk aversion, and thus people with low cognitive abilities show more risk aversion. Positive values indicate a positive correlation between cognitive abilities and risk aversion, so that people with low cognitive abilities show less risk aversion.

**Fig. 12:**
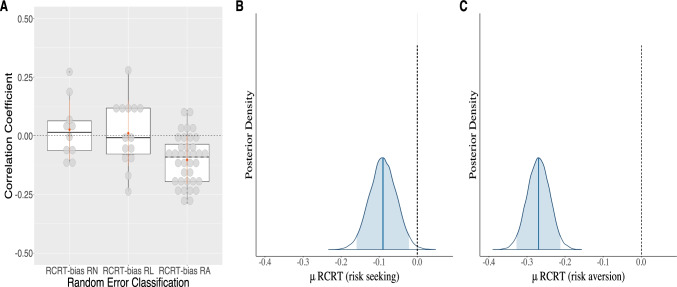
Raw data of the CRCT variable and its corresponding estimated posterior distributions. *Note.* Panel **a**: Raw data and mean difference of the correlation coefficients between risk aversion and cognitive abilities against the zero point (black dashed line). Shown are the proportion of choices in which the expected value (EV) of the less risky choice options is smaller (RCRT bias RA), larger (RCRT bias RL), or equal (RCRT bias RN) to the EV of the riskier ones. Panel **b**: The estimated posterior mean of the RCRT-bias variable for risk seeking (random error RS; i.e., the proportion of choices in which the EV of the less risky choice options is lower than the EV of the riskier ones). Note that the estimated posterior mean, indicated by a solid blue line in the middle of the distribution, does not differ from the zero point (indicated by the grey dotted line). The shaded area of the posterior distribution of the RCRT bias for risk seeking represents the 95% Bayesian credible interval. Panel **c**: The estimated posterior mean of the RCRT-bias variable for the risk-aversion (random error RA) variable (i.e., the proportion of choices in which the EV of the less risky choice options is larger than the EV of the riskier ones). Note that the estimated posterior mean, indicated by a solid blue line in the middle of the distribution, differs substantially from the zero point (indicated by the grey dotted line). The shaded area of the posterior distribution of the RCRT bias for risk aversion represents the 95% BCI. RCRT = Random choice risk taking

**Fig. 13:**
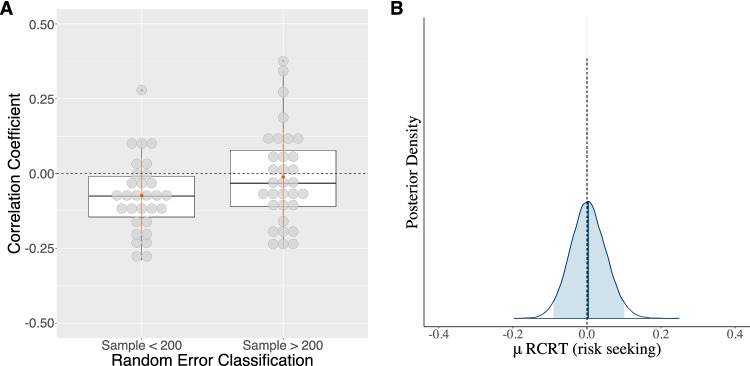
Raw data of the sample size (sample size >200) variable and its corresponding estimated posterior distributions. *Note.* Panel **a**: Raw data and mean difference between the groups against the zero point (black dashed line). Sample <200 and sample >200 refer to studies with less than 200 participants and more than 200 participants, respectively. Panel **b**: The estimated posterior mean for studies with *sample size* >200 (solid line) against the zero point (grey dotted line). Note that the estimated posterior mean, indicated by a solid blue line in the middle of the distribution, does not differ from the zero point (indicated by the grey dotted line). The shaded area of the posterior distribution of the sample size represents the 95% Bayesian credible interval

## Data Availability

Data and data analysis scripts are available on OSF (https://osf.io/m4qz9/).

## Appendix

**Table 6:** Linear models examined for explanatory variable selection procedure applying hierarchical regression procedure

Explanatory variable	*M*_*n*_	*M*_*f*_	*M*_*1*_	*M*_*2*_	*M*_*3*_	*M*_*4*_	*M*_*5*_	*M*_*6*_
Intercept	**−.03** [**−**.08, .02]	**.13** [.01, .26]	**−.004** [**−**.11, 11]	**.13** [.01, .25]	**.13** [.01, .25]	**.13** [.03, .23]	**.13** [.03, .24]	**.14** [.03, .25]
RCRT bias (risk averse)	—	**−.27** [**−**.33, **−**.21]	—	**−.27** [−.32, −.21]	**−.27** [**−**.33, **−**.22]	**−.27** [**−**.33, **−**.21]	**−.27** [**−**.33, **−**.21]	**−.27** [**−**.33, **−**.21]
RCRT bias (risk seeking)	—	**−.09** [**−**.16, −.02]	—	**−.09** [−.16, −.02]	**−.06** [**−**.12, .004]	**−.09** [**−**.16, −.03]	**−.09** [**−**.16, −.03]	**−.09** [**−**.16, −.02]
Type of cognitive test (CRT applied)	—	**.002** [**−**.04, .03]	**−.008** [**−**.04, .03]	**—**	**−.003** [**−**.04, .03]	**−.003** [**−**.04, .03]	**−.002** [**−**.04, .03]	**−.003** [**−**.04, .03]
Domain (potential loss included)	—	**.07** [.02, .11]	**.15** [.12, .19]	**.07** [.02, .11]	—	**.06** [.03, .11]	**.07** [.03, .11]	**.07** [.02, .11]
Type of choice options (sure-payment option included)	—	**−.03** [**−**.14, .09]	**.009** [**−**.10, .12]	**−.02** [−.14, .09]	**−.03** [**−**.15, .09]	—	**−.02** [**−**.14, .09]	**−.02** [**−**.13, .09]
Sample size (>200)	—	**.003** [**−**.09, .10]	**−.03** [**−**.12, .06]	**.002** [**−**.09, .09]	**.02** [**−**.07, .11]	**.01** [**−**.07, .1]	**—**	**−.0007** [**−**.09, .09]
Number of choice options	—	**−.0007** [**−**.003, .004]	**−.03** [**−**.12, .06]	**−.0007** [**−**.003, .004]	**.0007** [−.002, .004]	**.0002** [**−**.003, .003]	**.0006** [**−**.003, .004]	—
BF	**8.3×10** ^**34**^	1	**3.1×10** ^**25**^	**.04**	**4.43**	**.07**	**.11**	**.004**

*M*_*n*_*–M*_*6*_ are linear regression models. *M*_*n*_ is a random-intercept model. *M*_*f*_ is a full model that includes all recorded explanatory variables. The estimated posterior means are indicated in bold font. The 95% Bayesian credible intervals are indicated in square brackets. Dependent variables are correlation coefficients and the corresponding sampling errors. Explanatory variables are a dummy-coded explanatory variable for assessing risk-taking propensity in the case of random error, a dummy-coded explanatory variable for differentiating between standardized IQ tests and other tests for assessing cognitive abilities, a categorical explanatory variable representing domain with three levels (gain domain, loss domain, mixed domain), a dummy-coded variable for differentiating between elicitation methods consisting of a safe and a risky options versus elicitation methods consisting of two risky options, and a continuous variable representing the number of choices included in the elicitation methods. BF refers to the Bayes factor applied to compare between a full model and the respective model. CRT = Cognitive Reflection Test; RCRT = random choice risk taking

**Table 7:** Correlation table for moderator variables

Moderator variable	Domain (only losses included)	RCRT bias
Domain (potential loss included)	−1.0	−.27
RCRT bias	−.27	1.0

The correlation between the correlation coefficients associated with the domain and RCRT bias variables. RCRT = Random choice risk taking

**Table 8:** Linear models examined for explanatory variable selection procedure calculated for Lilleholt (2019)

Explanatory variable	M_n_	M_f_	M_1_	M_2_
Intercept	**−.05** [−.07, −.03]	**.06** [−.01, .14]	−**.04** [−.09, .00]	**.03** [−.05, .10]
Percentage of risk-averse options	—	−**.17** [−.25, −.09]	—	−**.12** [−.20, −.03]
CRT as cognitive test	—	−**.04** [−.09, .00]	−**.04** [−.08, .00]	−**.02** [−.07, .03]
Potential loss included	—	**.12** [.08, .15]	**.09** [.06, .11]	**—**
Sure-payment option included	—	−**.01** [−.06, .05]	−**.03** [−.08 .02]	**.03** [−.03 .09]
BF	**2.5×10** ^**12**^	1	**5.9×10** ^**6**^	**2.1×10** ^**10**^

*M*_*n*_*–M*_*2*_ are linear regression models. *M*_*n*_ is a random-intercept model. *M*_*f*_ is a full model, including all recorded explanatory variables. The estimated posterior means are indicated in bold font. The 95% Bayesian credible intervals are indicated in squared brackets. Dependent variables are correlation coefficients and the corresponding sampling errors. Explanatory variables are a continuous variable representing the percentage of risk-averse options, a dummy-coded explanatory variable for differentiating between standardized IQ tests and other tests for assessing cognitive abilities, a categorical explanatory variable representing domain with two levels (gain domain, losses included), and a dummy-coded variable for differentiating between elicitation methods consisting of a safe and a risky options versus elicitation methods consisting of two risky options. BF represents the Bayes factor calculated to compare the full model against each of the proposed models. CRT = Cognitive Reflection Test

Appendix Figure 11

### Complementary results

This section provides additional analysis to support the rationale of utilizing a multilevel approach by showing the consequences of including studies reporting a single effect size and those reporting multiple effect sizes. Additionally, we report the results of the RCRT-bias variable when defining it as a categorical variable. Last, we present the results of the role of sample size in moderating the link between risk aversion and cognitive abilities.

#### Variation between and within studies

To determine the necessity of a random-effect multilevel model for the current meta-analysis, we computed an intraclass correlation for Bayesian models. This method quantifies the proportion of variance that is explained by the grouping structure of the hierarchical model. In the case of an intercept-only model the computation of this proportion is as follows: First, a sample is drawn from the posterior distribution that originates from the variation between studies (i.e., from the posterior distribution of *μ*_*j*_) as well as from the posterior distribution that originates from the variation within studies (i.e., from the posterior distribution of *ϵ*_*ij*_). Second, the variance of those samples is calculated. Last, the ratio between the two variances is calculated following the formula:

$$Intraclass\ correlation=\frac{\mu_j}{\mu_j+{\epsilon}_{ij}},$$

where *i* represents the reported effects sizes and *j* represents the effects sizes nested within each study, *μ*_*j*_ is the random intercept drawn from a Cauchy distribution, and *ϵ*_*ij*_ represents the residual variance. The result of this analysis for the current meta-analysis is .71 with a 95% confidence interval ranging from .49 to .83. These results indicate that 71% of variance can be attributed to the between-study variation. Furthermore, this implies that there is a considerable level of variation within-studies. These results emphasize the importance of applying a suitable model, which can account for the variances on both levels, such as the random-effect multi-level models.

### Explanatory variables

#### RCRT bias

In this section, we report the results obtained by the inclusion of two moderator variables, the dichotomized threefold classification of the RCRT bias (see Method, Study Characteristics section). RCRT bias towards risk aversion tend to report a stronger negative correlation between cognitive abilities and risk aversion (see Appendix Table 8, Model *M*_*f*_ and see Appendix Fig. 12) with a mean estimated coefficient of −.27 was somewhat attenuated with a 95% BCI ranging from −.33 to −.21 (see Appendix Fig. 12a). As for studies applying risk-preference-elicitation methods with an RCRT bias towards risk seeking, the mean estimated coefficient of −.09 was somewhat attenuated with a 95% BCI ranging from −.16 to −.02 (see Appendix Fig. 12a). The attenuated effect for tasks with an RCRT bias towards risk seeking might be due to the low number of reported observations (*n* = 14). We calculated the raw mean effect size for those studies that applied a task architecture that fell under the RCRT categorization as (a) biased towards risk seeking: .01 (*SD* = .14) with a total of *n* = 14 reported correlations; (b) biased towards risk aversion: −.10 (*SD* = .10) with a total of *n* = 36 reported correlations; and (c) no bias: .03 (*SD* = .12) with a total of *n* = 10 reported correlations. We ran a linear Bayesian regression using random intercepts to test the role of the task-architecture variables in the reported correlation between cognitive abilities and risk aversion (see Appendix Table 6). Overall, studies with an RCRT bias towards risk aversion and risk seeking reported a substantial negative correlation between cognitive abilities and risk aversion (see Appendix Table 6, Model *M*_*f*_). Importantly, please note that the interpretation of these results should be taken with caution, since the clusters are not equal in size. This is particularly the case for the comparison against the reference group (i.e., the no-RCRT-bias group) as here the number of observations is relatively small.

#### Sample size >200

The role of sample size in the significance of the correlation between cognitive abilities and risk aversion was pointed out by Dohmen et al. (2018), who claimed on page 125: “*no statistically significant relationship is observed in studies that involve small sample sizes of less than 200 observations.*” Sample size can affect the chance to observe significant results. Simply put, as sample size increases so does the chance to observe a significant effect size. In the current analysis, we used the sample size as an indirect operationalization of significance and examined whether sample sizes change the relation between cognitive abilities and risk aversion. To do so, we coded the number of participants associated with each effect size. More specifically, we split sample sizes into two groups, one for sample sizes greater than 200 (value 1 assigned) and the other for sample sizes less than 200 (value 0 assigned). The inclusion of the sample size explanatory variable results in a mean estimate of .003 and a 95% BCI ranging from −.09 to .10 (see Fig. 9a). This estimate seems to be robust across all models’ specifications (see Appendix Table 6, Models *M*_*f*_*–M*_*6*_). Again, a model comparison procedure that weighs the evidence of a full model (see Appendix Table 6, Model *M*_*f*_) against a model without the sample size predictor suggests moderate evidence in support of the model without this variable (see Appendix Table 6, Model *M*_*5*_). We conclude that this variable is negligible in guiding the strength of the correlation between cognitive abilities and risk preferences.

Appendix Figure 13

## Footnotes

1 Past research has used similar terms, such as *risk taking*, *risk preferences*, *risk appetite*, or *risk attitude* when referring to people’s willingness to take risks. We consider people’s risk preferences as a latent cognitive construct. We will use the general term *risk preferences* when we refer to people’s risk-taking preferences and indicate the direction as *risk averse* or *risk seeking*. Furthermore, we use the term *risk taking* when referring to people’s observed behaviour, where increased risk taking refers to *more observed risk-seeking behaviour* and decreased risk taking refers to *more observed risk-averse behaviour*. Finally, for comparability, we use the correlation between cognitive abilities and *risk aversion* as our outcome variable, as most studies have reported results in terms of high or low risk aversion.

2 The European Commission issued the Markets in Financial Instruments Directive (MiFID) 2014/65/EU (see also MiFID II) and the Financial Industry Regulatory Authority in the United States issued Rule 2111. According to these regulations, investment firms are obliged to adapt their investment offers to the individual risk profile of the customer.

3 We consider risk preferences as a latent cognitive construct that can be inferred from several observable measures (e.g., behavioral or self-reported). For instance, `Frey et al. (2017) examined a large battery of risk elicitation methods. They showed that all measures share a common factor that emerges from individuals’ stated risk preferences. These results speak in favour of risk as a latent construct. Importantly, the authors further find high variability across all considered measures, in particularly, among behavioural tasks (see also Pedroni et al., 2017). The findings in the current work support this claim. We show that behavioural task architecture contributes to the variability across behavioural measures. The interaction between task architecture and individual characteristics (e.g., likelihood to choose randomly) can lead to a biased observation of the willingness to take risk. In fact, our proposed error hypothesis indicates that, given that risk is a latent construct and can be inferred from individuals’ behaviour, even if a given risk elicitation measure is biased, people with high cognitive abilities are likely to choose according to their risk preferences such that this observation provides a relatively accurate representation of this individual risk preference. In contrast, for people with low cognitive abilities, biased risk elicitation measures effect the inferred risk preferences due to the higher rates of random choice behaviour. This can result in a biased estimation of these individuals’ risk preferences.

4 Perceptual coding theory (`Frydman & Jin, 2018; `Khaw et al., 2020) provides another explanation for the potential link between cognitive abilities and risk aversion. This theory focusses on humans’ biased and noisy perception of smaller numbers compared with larger ones, which leads to a distorted mental representation of the decision problem. Accordingly, the same numeric distance appears larger in the mental representation of small numbers than of large ones. In risky decisions, such a mental representation of small-stake gambles compared with large-stake gambles decreases the likelihood of taking a risk, regardless of the actual risk preferences (`Khaw et al., 2020). Hence, given an identical choice set, if decision makers with higher cognitive abilities have a less biased mental perception than decision makers with lower cognitive abilities, they are expected to show more risk-seeking behaviour. Consequently, one should expect a negative correlation between cognitive abilities and risk aversion. Importantly, once lottery stakes increase, the difference in risk taking is predicted to decline as the bias in the mental representation becomes smaller.
